# The *mega-structure* at Stăuceni-‘Holm’, Botoşani county, Romania and the debate about the governing of Cucuteni-Trypillia-settlements

**DOI:** 10.1371/journal.pone.0343603

**Published:** 2026-03-27

**Authors:** Doris Mischka, Carsten Mischka, Adela Kovács, Constantin Aparaschivei, Elena Marinova

**Affiliations:** 1 Friedrich-Alexander-University Erlangen-Nürnberg, Institute for Pre- and Protohistory, Erlangen, Germany; 2 Botoşani County Museum, Botoşani County, Romania; 3 National Museum of Bukovina, Suceava, Romania; 4 Laboratory for Archaeobotany, Baden-Württemberg State Office for Cultural Heritage, Hemmenhofen, Germany; Austrian Academy of Sciences, AUSTRIA

## Abstract

2021 to 2024, the Cucuteni A3 settlement of Stăuceni-‘Holm’, Botoşani County in north-east Romania was surveyed geophysically and by systematic field collections. According to the geomagnetic results, on the plateau a settlement with about 45 houses was delimited by several ditch- and palisade systems. A comparatively large building (350 m^2^) was located in the area between the ditches, which is meant to be a mega-structure, mainly due to its size and the clearly visible position next to the probable entrance of the settlement. The mega-structure was partially excavated in 2023–2024. The observations, regarding the architecture and the dating of the feature in particular, provide valuable information for the discussion about the function of these special structures, of which only five others have been investigated in detail by excavation to date.

## 1 Introduction

Since the partially enormous spatial extent of the Cucuteni-Trypillia-settlements became visible by geophysical methods and aerial photography [e.g. [1–6]]; 6 with a resumé of the research history] the question arose, how it could have been possible to manage such quite large settlements without obvious hierarchical structures or wealth differences. Culminating in the mega-sites with at least 5.000–10.000 contemporaneous dwellings, sheltering perhaps 3–5 times as many people [e.g. [7,8]], some kind of social organization, regulating the economy and the social living, seems to be mandatory.

At first sight, most of the Cucuteni-Trypillia settlements, the mega-sites in particular, appear well planned and systematically arranged, without differences among the houses, without special buildings, markets or ritual infrastructure. Script and writing, useful for administrative tasks, is not yet invented or detected through the archaeological research. Perhaps the so-called tokens, little clay objects, could indicate some kind of counting system. However, the houses were interpreted as contemporaneous and reflecting the society behind the system as a kind of egalitarian or non-hierarchical, without “rulers” [e.g. [9–15]]. This was the case even after it turned out, that some of the mega-sites, like Maidanetske, were probably in use for about 350 years (3990−3640 cal BC (1ς) [16].

One may question, if this line of research is a good premise, regarding to methodological aspects, nevertheless, it is still used in recent times. So far, the layout of the ground plans and the different features are the only data we can take into account for a suggestion of political, social and economic organization, together with the data resulting from excavations providing more insight in the temporal development and functional interpretations of the find inventories from the excavated features.

Regarding the size of the sites under study, it seems impossible to excavate one site completely in order to get the full picture. But the modern research, with its improved technical possibilities for getting a closer look to construction details by surveys of entire settlements, test excavations and drilling programs, has brought to light that both the houses, and the settlement ground plans observed, occasional show differences among the buildings, their sizes and position.

This article, dealing with the large house-like constructions labelled mega-structures within Cucuteni-Trypillia settlements, aims to contribute with new evidence to the discussion of social and political organization of such sites, as well as the rise and the rejection of hierarchical systems [cf [4,11,17–19]].

So far, the mega-structures were interpreted as communal facilities for decision-making or other purposes [4,11,18].

In the summers of 2023 and 2024, four-week excavation campaigns with students of the Institute of Pre- and Protohistory of the Friedrich-Alexander-University of Erlangen-Nürnberg, together with the Botoşani county museum, took place at Stăuceni-‘Holm’, jud./county Botoşani. The first important, but still preliminary, results are published here because they shed new light on the phenomenon as such.

The research presented here targets **the type of architecture** of these mega-structures, **their dating** and comparison to other structures excavated, as well as **their function within the settlement** and the society using them.

## 2 Mega-structures

**Fig 1:**
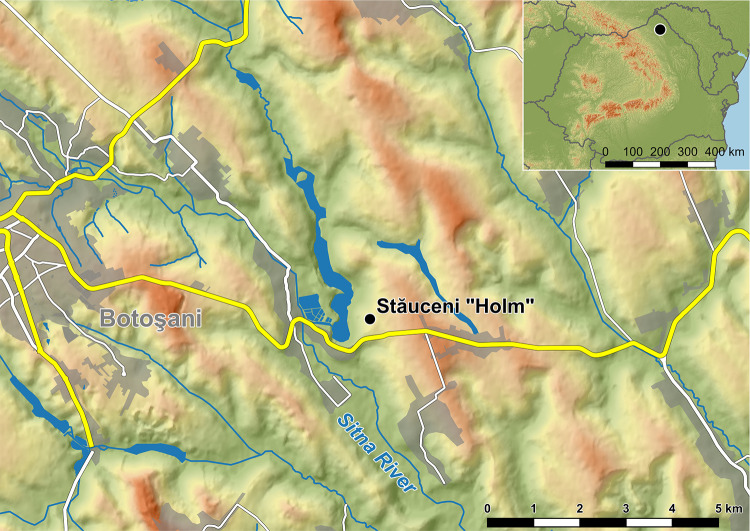
Stăuceni-‘Holm’. **Location of the site within the Sitna valley, east of Botoşani, Romania.** Made with QGIS. Own creation (C. Mischka), using SRTM [20] and OSM Data. OSM: Map data copyrighted OpenStreetMap contributors and available from https://www.openstreetmap.org.

In this article, we focus on the site Stăuceni-‘Holm’, situated in Botoşani county in northeastern Romania (Fig 1). Geomagnetic survey of the site revealed the ground plan of a settlement, showing plots of burned houses, with mostly the same size. However, two house plots are significantly larger, so they can be labelled as “mega-structures” according to Видейко and Chapman [21,22], who named their findings from geomagnetic surveys in Nebelivka as such. Hofmann et al. analysed 140 mega-structures from 19 sites, mainly in Ukraine and the Republic of Moldova [19]. Their compilation of the state of research and their ideas of the function of mega-structures are still valid.

Mega-structures are in the meanwhile defined as follows [13,19,23] or [24]:

Mandatory:

- Rectangular constructions bigger than normal houses
- Located mainly in free spaces, such as ring-corridors or so-called plazas
- Located in highly visible positions

Additionally:

- Different architecture than domestic dwellings
- Extraordinary dimensions

Ohlrau distinguishes between mega-structures of up to 70 m in length and up to 24 m in width, occurring only singularly at “mega-sites” and the “ring- or pathway mega-structures” [16].

For the authors of this article, the *relative* difference in size compared to the other buildings in the settlement is particularly decisive for the assessment of a mega-structure. In the Linear pottery culture for example, houses with dimensions up to 35 x 10 m are nothing extraordinary and not labelled as mega-structures [25]. The relevant feature in Stăuceni-‘Holm’ with around 350 m² is a significant outlier, compared to the site’s other house plots with a size of approximately 70–120 m² (interquartile, median: 91 m²). In addition, the clearly visible position, directly behind the ditch, presumably near the entrance, speaks in favour of the interpretation as a mega-structure.

## 3 State of Research on Mega-structures

**Fig 2:**
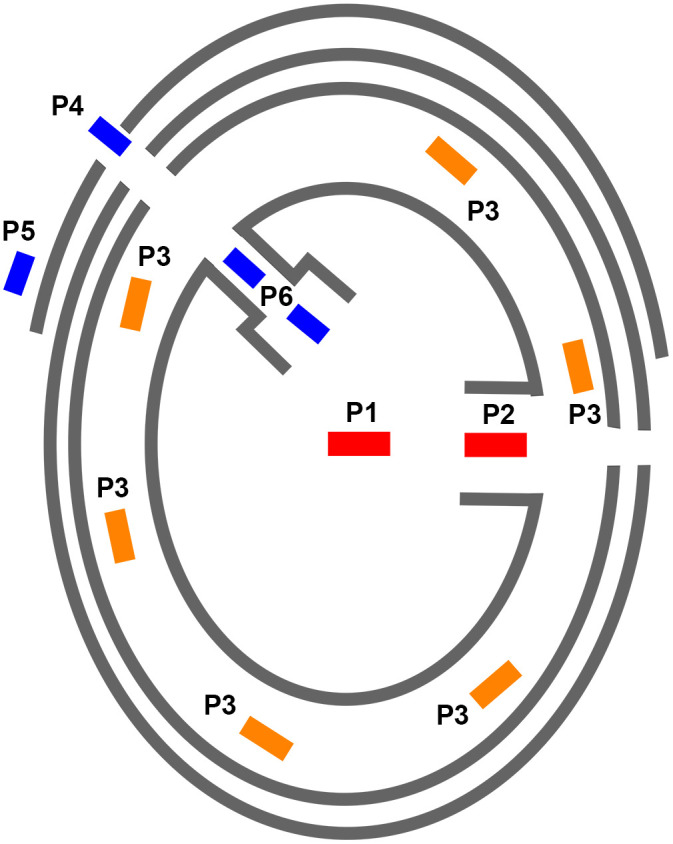
Schematic plan of the location of mega-structures in Trypillia ring settlements and mega-sites according to Hofmann et al. 2019, 36-37 Fig. 19 [19]. https://journals.plos.org/plosone/article/figure/image?size=inline&id=10.1371/journal.pone.0222243.g019.

The relevant results from the paper of Hofmann et al. 2019 [19] are:

- The mega-structures occur within the ring-settlements of the Trypillia culture on six different positions: P1 at a primary central place next to the presumed main entry from the eastern direction into the mega-sites, P2 in secondary places, P3 along the ring-corridor, in the radial pathways close to the outer rim (P4) or closer to the centre of the settlement (P6) or at the outside of the circle (P5) [19]; Fig 2).
- Their location within the settlement correlates with their dimensions [19].
- There are tempo-spatial differences in their layout, size and position [19].
- A typological classification is possible by dimension, internal division and internal features [19].

The internal divisions and features are of special interest here. Among the excavated mega-structures, they play a key role within the discussion if only part of the structures were roofed and other parts were “largely open structure surrounded by wattle-and-daub walls whose destruction daub has remained in place” [26]. The interpretation of a (partly) non-roofed courtyard is based on the quite low amount of daub, which is also often not as much heated as at the burnt domestic dwellings. Furthermore, installations of fireplaces are less frequent and ovens are completely missing. Also, the quantity and quality of all kinds of finds is low in comparison to the smaller so-called domestic houses.

Within the geomagnetic plans, the low amount of burnt daub seems to be visible as well. Here, mega-structures with a lot of high susceptibility records within the features can be divided from those, showing only higher values along the supposed outer walls. This is interpreted by Hofmann et al. according to the excavations as indication of roofed versus un-roofed or only partly roofed constructions [19,26].

Furthermore, Hofmann et al. compare the floor area of mega-structures with ethnological data of communal structures [19]. Therefore, they calculate the size of the user group by dividing the number of normal dwellings by the number of mega-structures, and estimating the number of inhabitants within the estimated contemporaneous houses. The ethnographic sample distinguishes between low-level and high-level special buildings, both of which can be the case for the Cucuteni-Trypillia mega-structures, according to the typological differences. The basic idea is that, in order to form a mega-site, a certain number of mega-structures constitutes the communal centre of up to 150 neighbouring households, and, via these low-level mega-structures, the high-level mega-structures, found mainly on the central plaza (P1), manage the entire settlement. For Nebelivka, Gaydarska et al. are seeing the mega-structures as part of quarters consisting of more than ten neighbourhoods of 3–7 up to 27 houses, so 30–270 houses belong to one mega-structure [26]. There is an overlap for the size of the user groups between high-level ethnographic and the low-level Cucuteni-Trypillia settlements, but the high-level Cucuteni-Trypillia mega-structures are far beyond the registered ethnographic upper limits. Thus, as mentioned by Hofmann et al. [19], the number of estimated inhabitants by persons/square meter is too big. An interpretation would be that the increasing user group size indicates the way to some kind of concentrated power within the communal structures by fewer persons and therefore more hierarchical structures with the disappearance of the low-level mega-structures. This would mean that the Cucuteni-Trypillia population of a mega-site was able to cover-up with user groups bigger than ever. This could be one of the factors leading to the end of the mega-sites, because of the non-acceptance of the new higher-ranked individuals or sub-groups among the inhabitants [19].

### 3.1 (Partly) excavated mega-structures

**Fig 3:**
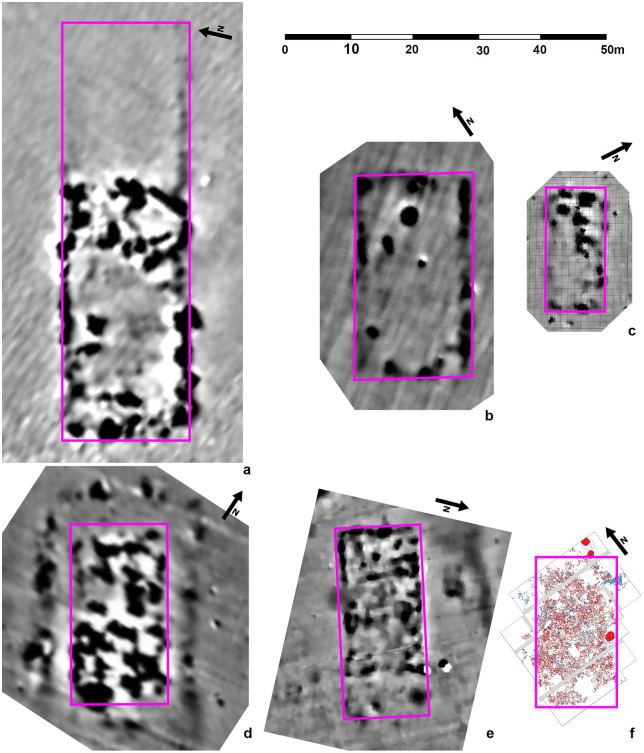
Magnetograms or excavation plan of the sites with excavations in mega-structures. A – Nebelivka, Kirovohrad oblast/Ukraine, b – Dobrovody, Cherkasy Oblast/Ukraine, c – Maidanetske, Cherkasy Oblast/Ukraine, d – Ripiceni-‘Holm’/La Telescu, Botoşani county/Romania. e – Stăuceni-‘Holm’, Botoşani county/Romania, f – Baia-*În Muchie*, Suceava county/Romania. Data taken from: a) [27] doi.org/10.1515/9783110664959, b) own work, c [19] https://journals.plos.org/plosone/article/figure/image?size=inline&id=10.1371/journal.pone.0222243.g004, d [28] https://pub.mdpi-res.com/remotesensing/remotesensing-12-00887/article_deploy/html/images/remotesensing-12-00887-g005.png?1584550918, e) own work, f) “Reprint from [29] under a CC BY license, with permission form [Emil-Constantin Ursu], original copyright [original copyright 2015]”.

**Fig 4:**
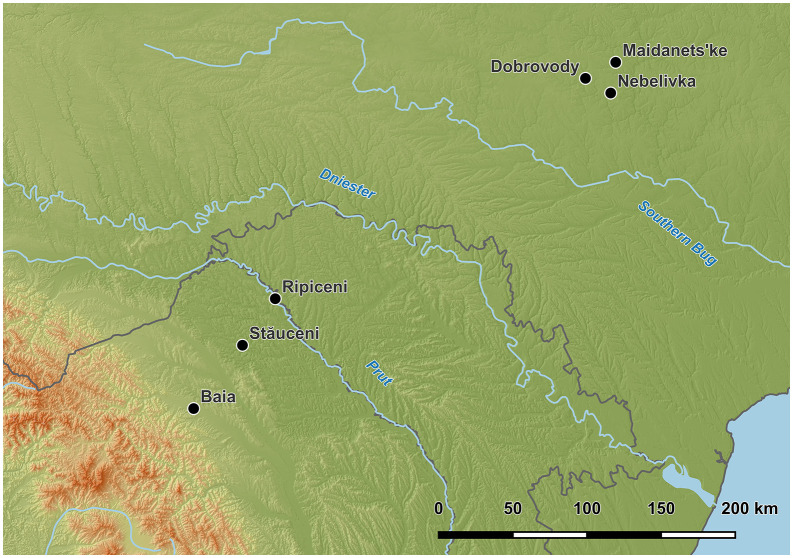
Map of the sites with compared mega-structures. Nebelivka Kirovohrad oblast/Ukraine, Dobrovody, Cherkasy Oblast/Ukraine, Maidanetske, Cherkasy Oblast/Ukraine, Ripiceni-‘Holm’/La Telescu, Botoşani county/Romania, Stăuceni-‘Holm’, Botoşani county/Romania, Baia-*În Muchie*, Suceava county/Romania. Made with QGIS. Own creation (C. Mischka), using for raster data SRTM [20] and OSM data for borders and rivers. OSM: Map data copyrighted OpenStreetMap contributors and available from https://www.openstreetmap.org.

The study of Hofmann et al. [19] is built nearly exclusively upon the interpretation of geomagnetic surveys, as so far only five mega-structures were excavated. The oldest one, **Baia - *În Muchie****,* Suceava county/Romania is dated to Pre-Cucuteni I period and is dated approximately to 5000 BC [11,13,26] (Figs 3 and 4). The mega-structure lies in the centre of the settlement. Within a so-called foundation ditch huge posts with pits more than 1 m deep were used to build the outer limits. In Baia more than 200 pots, some of them with anthropomorphic representations, and a lot of other finds have been registered within the building of about 220–230 m^2^ [11,13,29].

**Nebelivka,** Kirovohrad oblast/Ukraine is dated between 3990/3840-3850-3740 BC (Trypillia BII) with a use span of maximum 160 years [4,22,26,30–32 argues for at least 200 years)] (Figs 3−4). The feature consists of two parts with lengths of 20−24 m and 38−40 m. The western part is characterized by a huge amount of burnt daub. The eastern part instead is only surrounded by a strip of 1–1.5 m width of burnt daub fragments, while the internal part is nearly empty of finds. Further, this eastern part is about 0.4 m lower than the ground floor of the western part [30]. After removing 0.5–0.6 m of sediment at the eastern part, no features came to light. Chapman et al. note: „Excavation to a greater depth also confirmed no obvious ditch profiles” [22] page 140. Because of the excavation technique, they could not exclude that a ditch once existed, because of a “removal through over-excavation” [22] page 140, whatever that means. At the western part, four phases could be differentiated according to stratigraphy: a pre-mega-structures phase with pits and postholes, a two-stage use phase, and a post-phase with destructions and re-fillings [26]. Altogether, the mega-structure covered 1120 m^2^ with dimensions of 60.5 x 18.3 m.

Large platforms, substructions for fireplaces were found, which can also be found in ordinary houses, but much smaller in size. Also, decorations occur in connection to the platform of the *mega-structure*.

Altogether, more than 60 kg of potsherds, coming from at least 332 pots were discovered. Special finds are limited to a small fragment of gold, a bowl with graphite decoration connected to the Balkan, and a set of miniature vessels [22,33], nor the quantity is different to the small houses. Only six flint artifacts were found, among them one arrow-head [25,34], with the small number not surprising, given the long distance to the nearest flint sources.

The mega-structures are understood here as roofed assembly houses or even temples, depending on the different opinions of the archaeologists, with a connected courtyard or “fenced” space [22,30] with the different interpretations of the mega-structure excavated at Nebelivka; [31]. Gaydarska et al. interpret the mega-structure at Nebelivka as “(…) what remains a public building but one without the depositional characteristics of a ritual or administrative centre” [34] page 211] with monumental character and “which created the potential for major congregations of several hundred people within the open courtyard in the Eastern part and the inner Central area of the Western part. The multiplicity of platforms was a result of the varied social groups (clans or lineages rather than families) participating in Mega-structure ceremonies. The destruction of the Mega-structure by fire would have been one of the great ceremonial manifestations” [34] page 211–212]. Korvin-Piotrovskiy prefers instead a complex of related households or alternatively a ritual complex [35].

The mega-site of **Maidanetske**, Cherkasy Oblast/Ukraine, covers 170 ha with 2930 houses counted [8] (Fig 3, 4). Eye-catching is a 100 m wide ring-road at the site, in which several mega-structures are placed at more or less equal distance to each other. Altogether, 13 mega-structures of different sizes are integrated in the settlement (small-size mega-structures of 150–200 m^2^ and medium-size ones of 200–300 m^2^). Several of them are built within the ring-roads, in particular large ones with dimensions of more than 575 m^2^ [e.g. [7,8,13]]. Others are oriented parallel to the road and orthogonally to the circular house rows.

The site belongs to Trypillia BI, Tomashivka local group, and is dated to 3990−3640 cal. BC [16]. The excavated mega-structure number 3, within the northeastern area of the ring-corridor, is dated most likely to the 38^th^ century BC [19].

The mega-structure measures only 19 m x 10 m [19]. Under it, the archaeologists found several pits which they connect to older houses. According to the distribution of burnt clay, they propose a roofed and an unroofed part of the construction. Using the find distribution patterns, they also propose different in- and outdoor activity zones [19].

For Maidanetske, it was proposed to consider the mega-structures as part of a quarter, consisting of 50–150 households and the social system interpreted in lineages and supra-household economic units [19,36].

Further at **Dobrovody,** Cherkasy Oblast/Ukraine, in a 150–250 ha mega-site, a mega-structure of 26 m x 46 m (1196 m^2^), located in a ring-corridor, was researched by a small survey of 144 m^2^ [5,19,34,35,37,38] (Figs 3, 4). The feature is dated to 3890–3720 cal. BC [39] and belongs to the second phase of the Tomashivka local group of Trypillia B2 [38,39]. Because of the low amount of daub, and also the low quantity of other finds, this feature is interpreted so far as “unroofed enclosure for unspecified ‘socio-economic purposes’” (according to Hofmann et al. [19]). Further, it appears to be parallel to the long side rows of several large, pit-like anomalies [5].

The site of **Ripiceni-‘Holm’/La Telescu**, Botoşani county/Romania is to a great extent already destroyed by the river Prut and the Stânca-Cost󠅣eşti water reservoir. 5.2 ha are preserved so far, but the erosion continues rapidly [28]. The settlement is partly formed by concentric rows of house plots [39,28]. It is dated by pottery to the Cucuteni AB1-phase [28]. From the mega-structure, a ^14^C-date is published as S.RIP.2: Poz-84451 5210 ± 40 BP; 4041−3975 cal. BC (1 sigma) [40]. The mega-structure was first interpreted as consisting of several non-cotemporaneous normal houses, partly overlapping at the same spot, only giving the impression of one very big structure in the geomagnetic plan [38]. It is situated in front of an inner ditch system delimiting the settlement’s core. The mega-structure measures about 38 x 19 m/ resp. 722 m^2^, according to the published geomagnetic results, Asăndulesei et al. giving a size of about 1000 m^2^ [28]. According to the geomagnetic plan, at the southeastern corner, the geomagnetic measurements show less strong anomalies which could indicate an entrance [28]. A strong anomaly is in front of that corner, and further south of the mega-structure no more burnt houses are visible, keeping a free view on the mega-structure for people approaching from this direction. West of the mega-structure, an area with clear anomalies could indicate one or even two more mega-structures or big houses, flanking the potential main entrance into the settlement. The mega-structure itself consists of a core part visible as a rectangular anomaly and surrounding linear features. This “enclosure”-anomaly is not visible in the south.

After the discovery in the late 1960’s, the site is under excavation since 2010 [41]. The mega-structure has been researched so far by five parallel trenches of 3 m width and 35 m in length, between 2016 and 2023 [40]. The trenches are cutting the mega-structure and the surrounding anomaly. Unfortunately, no ground plans or drawings of plans and sections are published so far and it is not easy to locate exactly the described features and photos, and to follow the descriptions of the reports. Nevertheless, the mega-structure seems to be very well preserved. A lot of finds are reported, as well as internal installations like ovens, grinding installations and internal walls. The archaeologists think there was an upper floor, built like an open-air terrace without roof [41]. The inner part is described as composed by several rooms, of which some were used for living, others for cooking or storing and, due to the find of a statuette, for cultic purposes as well. According to the descriptions, it is not clear if the archaeologists found a ditch system or a wall. This may be a construction similar to the one from Baia or Stăuceni-‘Holm’, but with a lot of finds found in the ditch, which is said to come from the mega-structure [41].

## 4 The site of Stăuceni-‘Holm’

### 4.1 Research history

According to the site sheet, the Cucuteni culture settlement from Stăuceni-‘Holm’, Stăuceni commune, Botoşani County was discovered by the well-known Romanian archaeologist Ion Nestor in 1952 (http://ran-tmp.cimec.ro/sel.asp?descript=stauceni-stauceni-botosani-asezarea-cucuteni-de-la-stauceni-dealul-holm-cod-sit-ran-35893.01). No further research followed, until in 2019, after agricultural activities in the spring, ceramic fragments typical of phase A3 of the Cucuteni Culture were identified on the ground by Adela Kovács from the Botoşani County Museum. After this new discovery, systematic surveys including geophysics started in 2021 and a first excavation was made in 2023.

### 4.2 Topography

**Fig 5:**
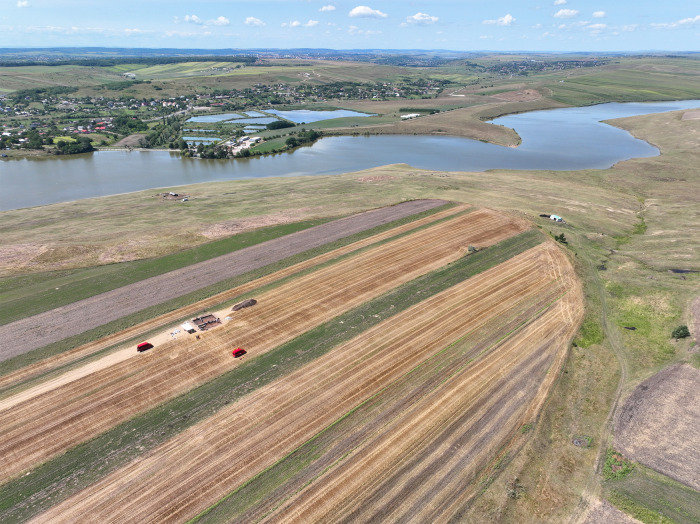
Stăuceni-‘Holm’. **Aerial photograph of the promontory with the reservoir at the bottom of the slopes.** Visible: The trenches of the 2023 excavation, cutting the southwestern corner of the “mega-structure” house number 5/6 (Fig 8). Aerial photography from drone, made by the author (C. Mischka).

The site Stăuceni-‘Holm’ is situated 6 km east of the city of Botoşani, on the north-eastern slope of the Sitna river valley in the forest steppe region (Fig 1). A modern water reservoir lies at the promontory’s foot. (Fig 5). The plateau of approx. 4 hectares is densely settled with at least 45 buildings from Cucuteni era. Along the eastern rim, heavy erosion affected the preservation of the features, shown by a multitude of ploughed-up surface finds.

### 4.3 Geophysical Surveys

**Fig 6:**
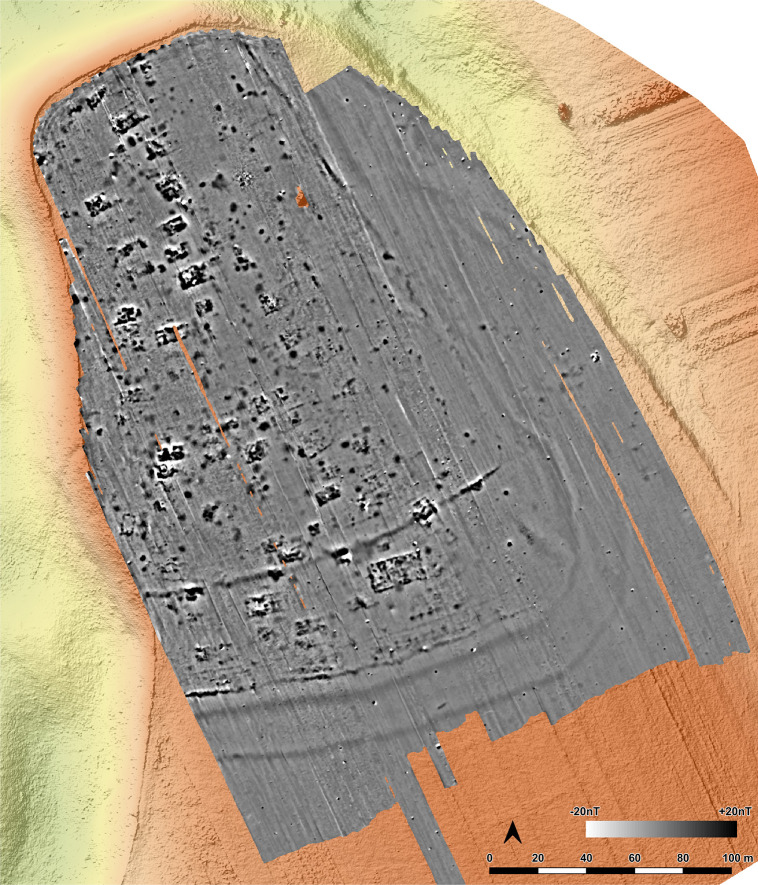
Stăuceni-‘Holm’. **Magnetogram of the site.** Made with QGIS. All used pictures made by the author (C. Mischka) and from data acquired/collected by the author (C. Mischka). – Magnetogram. - Digital surface model made by SfM and Drone.

**Fig 7:**
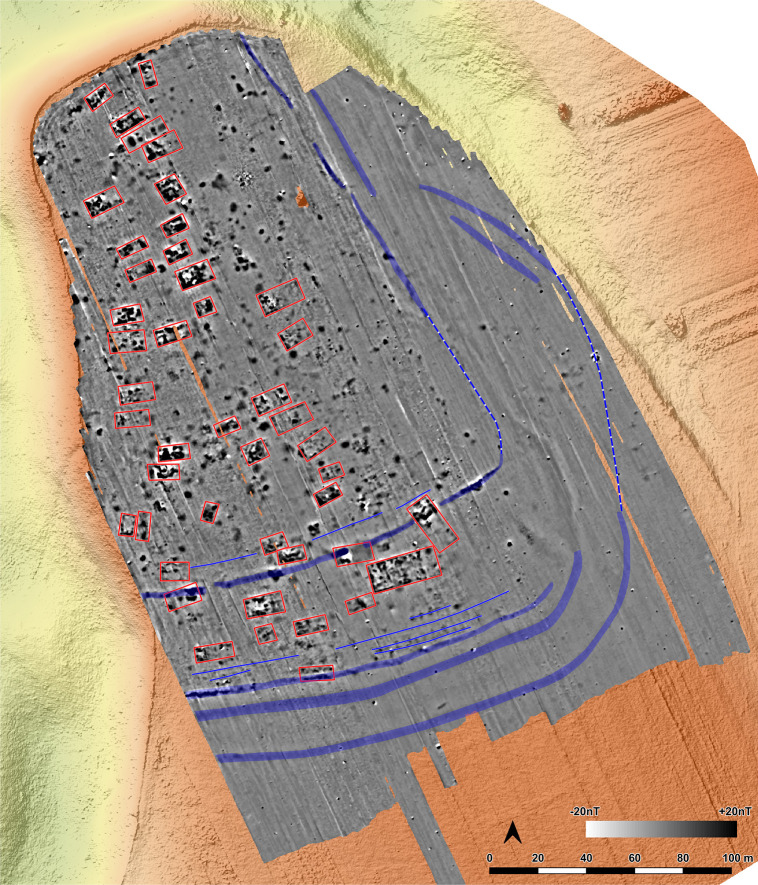
Stăuceni-‘Holm’. **Magnetogram of the site with indication of the features.** Labelling of the features see Fig 8, explanation in the text. Made with QGIS. All used pictures made by the author (C. Mischka) and from data acquired/collected by the author (C. Mischka). – Magnetogram. - Digital surface model made by SfM and Drone. - Archaeological interpretation layer.

**Fig 8:**
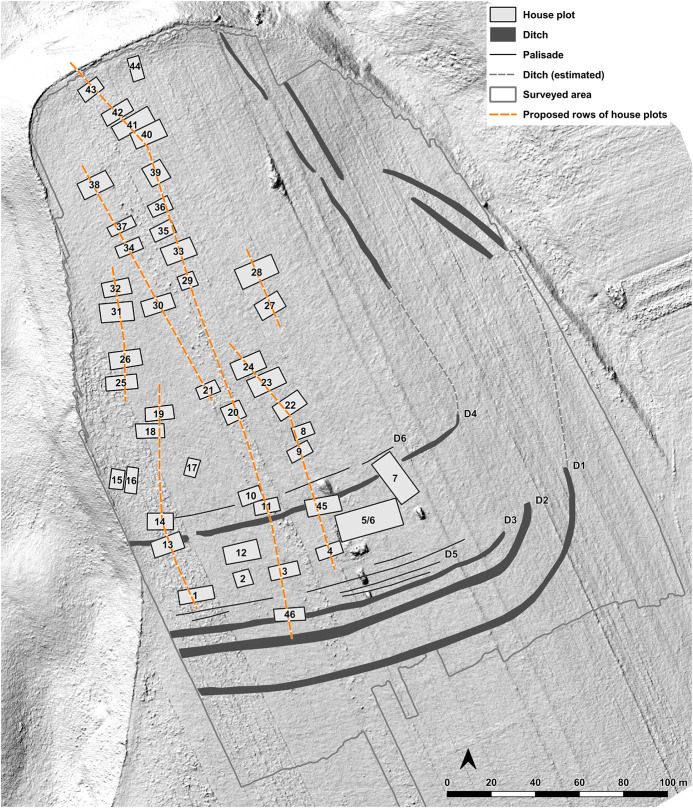
Stăuceni-‘Holm’. **Plan of the labelled features of the settlement according to the magnetogram (Figs 6**, **7).** Made with QGIS. All used pictures made by the author (C. Mischka) and from data acquired/collected by the author (C. Mischka). - Hillshading of digital surface model made by SfM and Drone. - Archaeological interpretation layer.

In 2021 and 2022 the site was surveyed with a gradiometer (Sensys-DLM 98 five probes with RTK-DGPS coupling) (Figs 6–8). The detected anomalies can be classified and grouped into different features, which can be interpreted as follows according to excavations at other places.

#### 4.3.1 Ditch systems (Figs 6–8).

Towards the south and east, a system of three partly parallel ditches (D1, D2 and D3; Fig 8) delimits the settlement. The ditches D2 and D3 run approx. 5m apart, while the outer ditch D1 runs approx. 18m further south. Another ditch (D4) surrounds the inner settlement, approx. 48m north of D3. It shows a nearly rectangular shape, similar to Scânteia, Iaşi county/Romania [42–44]. Further, several narrower (<1m) ditches (D5 and D6) parallel D1-D4 and can be interpreted as palisades, located only some meters behind the outer ditch system D1-D3 (D5) and behind the inner ditch D4 (D6).

#### 4.3.2 The buildings (Figs 6–8).

North of the ditches, the magnetogram shows 5–6 slightly parallel rows, made up by 4–14 houses in gable-topped arrangement (Fig 8 orange lines). More to the east, the houses are most likely already eroded, because of the steep slope, as a high amount of pottery sherds and adobe (daub) on the surface indicate. At the western and northern end of the promontory, some houses (numbers 15–16 and 44) are not totally fitting into this system and slightly turned diagonally to the edge of the plateau or longitudinal towards it.

In the southern area of the settlement, the houses are oriented parallel to the ditch systems (D1-D6), with one exception (number 7), crossing ditch D4, as do two other buildings (number 45 and number 13) following the ditch towards west. Ditch D3 is cut by an anomaly, possibly a house (number 46) as well. The overlaps prove at least two phases of the site. Also, in the northern part of the site, one anomaly which could result from a north-south-oriented building (number 44) could belong to another phase.

Between the ditches, some houses (numbers 2–4) are small, but others are bigger than the houses (number 7, also No. 12–13 and No. 45) within the delimiting ditches. With about 35 x 10 m (~350 m^2^), one feature (number 5/6) is clearly oversized, but following the main buildings’ orientation. With the exception that it is not positioned in a ring-corridor – which of course does not exist in a not ring-shaped settlement plan –, it fulfils all the requirements to be called “mega-structure” according to the definition mentioned in section 2.

#### 4.3.3 Pits (Figs 6, 7).

As normal for Cucuteni settlements, the complete site is covered with anomalies from settlement pits. Some linear arrangements could indicate further buildings, perhaps not burned or only weakly burned and therefore not so visible in the magnetogram.

### 4.4 Field surveys

In 2021–2023, intensive collection of surface finds took place to find out, if it is possible to get a more precise dating of the various structures, the houses within the ditch system and those placed orthogonally to those close to the ditches in the southern part of the site. The artefacts were collected using 8 m perimeters around the house plots and a 10 x 10 m grid in the areas without geomagnetic features. The processing of the data is still in progress, but the collected ceramic material comprises several boxes, especially from the grid area. This, together with the fact that almost none of the sherds have any traces of their initial painting, proves the high degree of destruction of the subterranean archaeological layers by agriculture. Also, 30 fragments of anthropomorphic statuettes were collected, some of them already being published in a specialized catalogue [45]. A full paper presenting the surface discoveries is in progress.

### 4.5 Excavation 2023–2024

**Fig 9:**
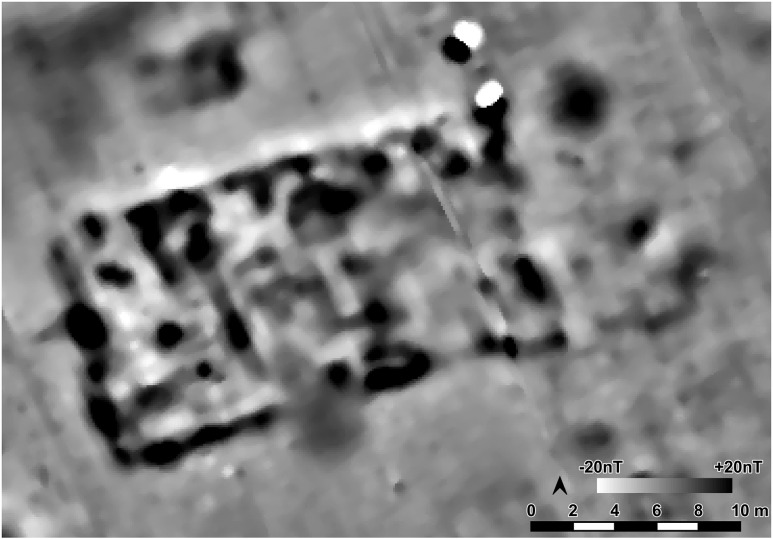
Stăuceni-‘Holm’. **Magnetogram of the house plot 5/6, the so-called mega-structure.** For the location see Figs 6–8. Made with QGIS. All used pictures made by the author (C. Mischka) and from data acquired/collected by the author (C. Mischka). - Magnetogram.

In 2023 and in 2024, a four-week excavation took place at the large House plot 5/6, the so-called *mega-structure* in the southern part of the site (Figs 6–9).

#### 4.5.1 Research objectives.

The extraordinary big anomaly is comparable to others mentioned above, i.e., the one at Drăguşeni-Ostrov, Botoşani county/Romania [46]. So far, the mega-structure in Stăuceni-‘Holm’ is located at the south-western edge of the phenomenon. The research objectives are therefore connected to the dating of the feature.

Our hypothesis is, that the mega-structure at Stăuceni-‘Holm’, dated preliminarily to Cucuteni A3, according to the survey pottery finds, belongs to the oldest mega-structures. If this is the case, it could be discussed, whether the concept of a mega-structure as a governing element evolved in the Romanian Cucuteni-region, from where it was distributed to the entire Cucuteni-Trypillia-area towards the steppe regions in the northeast.

The second objective concerns the function of the mega-structure. For the Ukrainian features, interpretations as assembly hall [16,31] or communal building [19,47] or as structure with ritual function [34,35] are proposed. At Maidanetske, Müller et al. and Hofmann et al. [17,19] describe, according to the site’s genesis, the “decline of medium sized mega-structures and the establishment of one large mega-structure” at later phases. Ohlrau et al. found out, that the mega-structures are present also in settlements smaller than mega-sites, which means that the buildings are essential parts of the settlement layout and structuring within the entire Cucuteni-Trypillia phenomenon and not related to mega-sites alone [16,23].

#### 4.5.2 Methodology.

**Fig 10:**
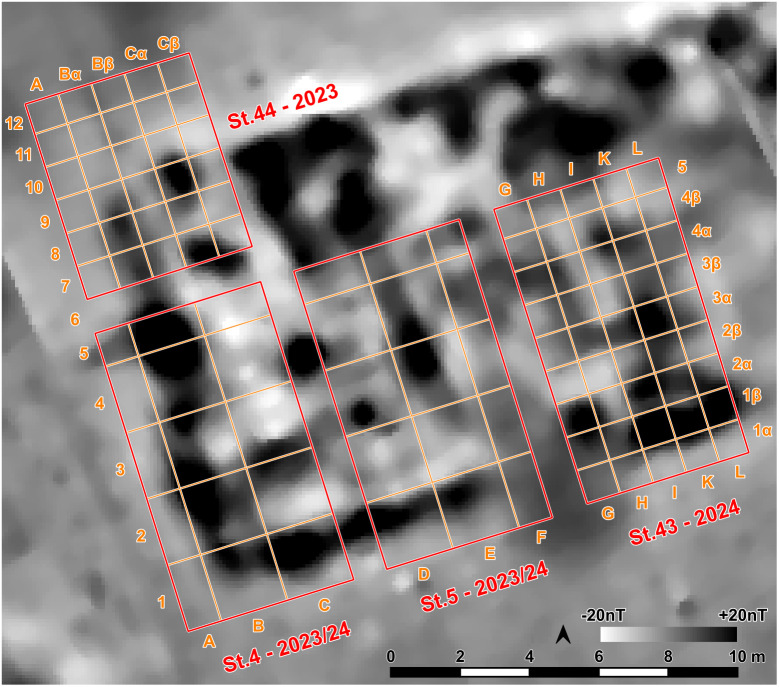
Stăuceni-‘Holm’. **Magnetogram of the mega-structure house 5/6 with indication of the excavation trenches and the grid system.** Made with QGIS. All used pictures made by the author (C. Mischka) and from data acquired/collected by the author (C. Mischka). – Magnetogram. - Layer with excavation grid.

**Fig 11:**
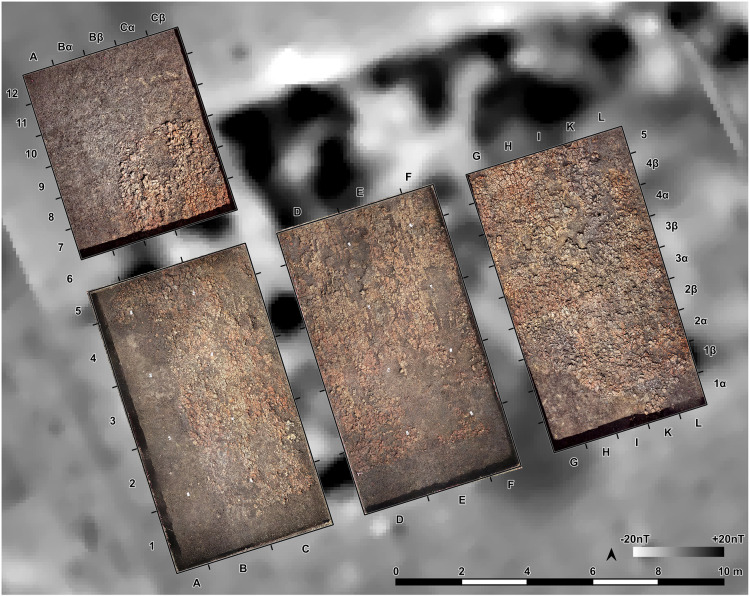
Stăuceni-‘Holm’. ***Mega-structure* house 5/6. Orthomosaic of the surface of the floor screed made of burnt clay.** Made with QGIS. All used pictures made by the author (C. Mischka) and from data acquired/collected by the author (C. Mischka). – Magnetogram. - Orthomosaics of excavation plana.

**Fig 12:**
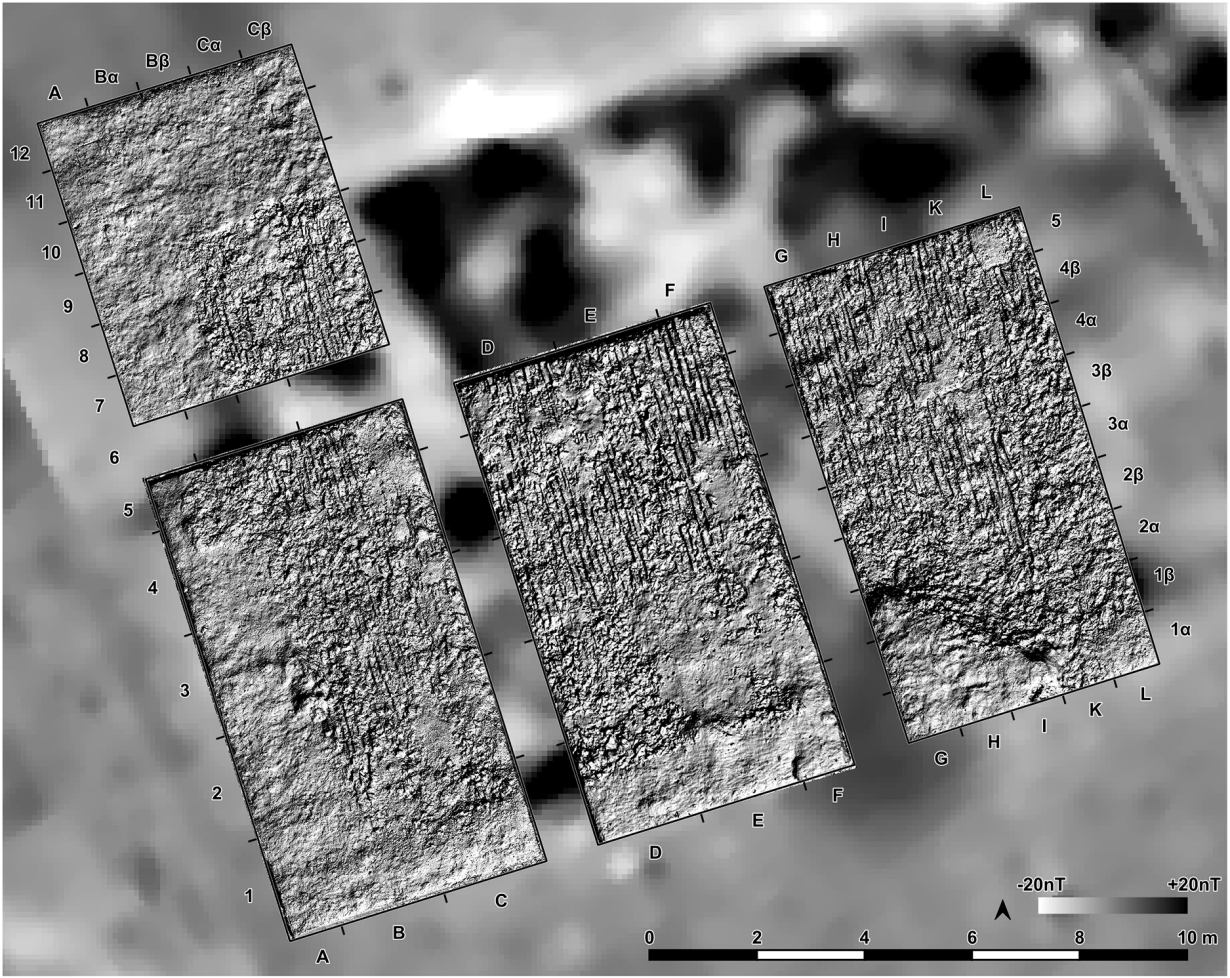
Stăuceni-‘Holm’. **Mega-structure house 5/6. Orthomosaic of the floor after removal of the burnt clay.** Visible: The imprints of the former wooden logs used as substruction for the clay. Made with QGIS. All used pictures made by the author (C. Mischka) and from data acquired/collected by the author (C. Mischka). – Magnetogram. - Hillshadings of digital surface models of excavation plana.

**Fig 13:**
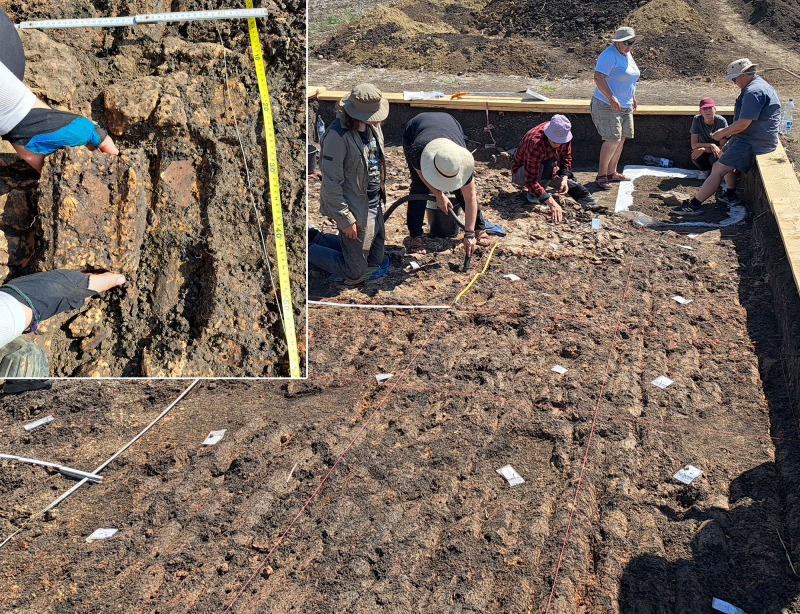
Stăuceni-‘Holm’. **Mega-structure house 5/6. Photo of a detail of the floor’s substruction.** Wooden logs were covered by loam which burnt and preserved thereby the shape of the wooden parts **as imprints in the earth.** All used pictures made by the author (C. Mischka): – two photographs of excavation motives.

The southwestern corner of the mega-structure was excavated with four trenches in 2023–2024, labelled St. 4, St. 5, St. 43 and St. 44 (Fig 10). The excavation was made mainly in natural layers, and if necessary, with additional artificial plana. The first plan shows the surface of burnt clay (daub) (Fig 11). The burnt daub covered decayed wooden logs of which only the imprints in the burnt daub remained (Figs 12, 13).

**Fig 14:**
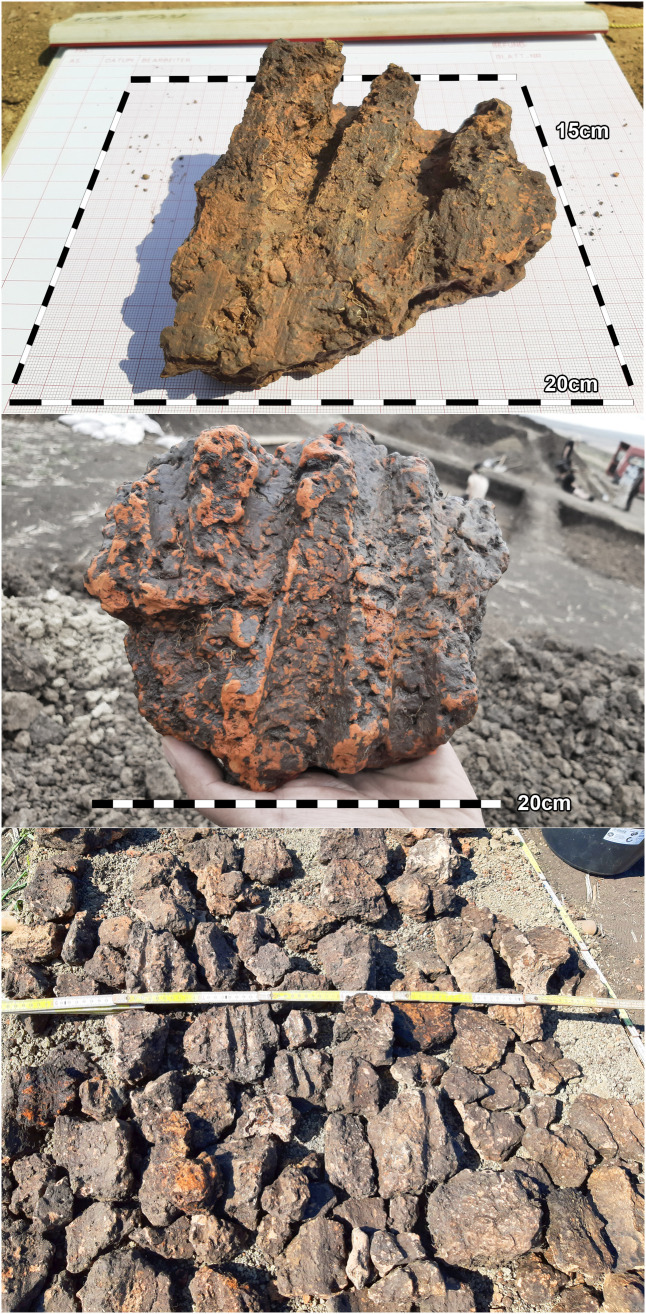
Stăuceni-‘Holm’. **Mega-structure house 5/6. Photo of burnt daub from the eastern end of trench 43 showing imprints of twigs or boards.** All used pictures made by the author (C. Mischka): – three photographs of excavation motives, two with added scalebars.

**Fig 15:**
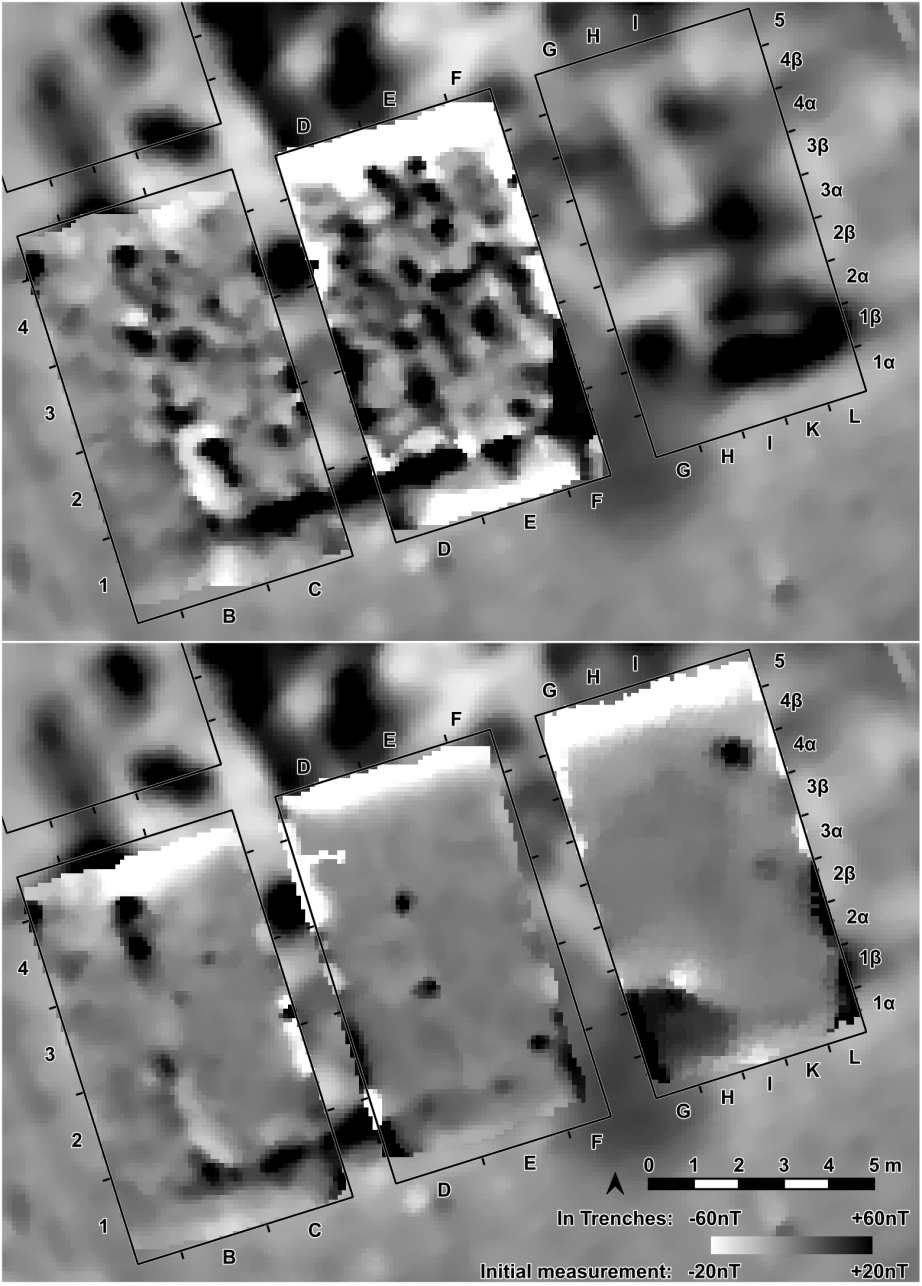
Stăuceni-‘Holm’. **Mega-structure house 5/6. Geomagnetic measurements within the excavated trenches St. 4-5 and St. 44.** Top: On the level of the floor imprints – Visible: The foundation ditch, but still disturbed by burned clay fragments. Bottom: Approximately 30 cm underneath the level of the floor imprints. Clearly visible: Foundation ditch and possible post holes. Made with QGIS. All used pictures made by the author (C. Mischka) and from data acquired/collected by the author (C. Mischka). – Magnetogram. - Layer with excavation grid.

**Fig 16:**
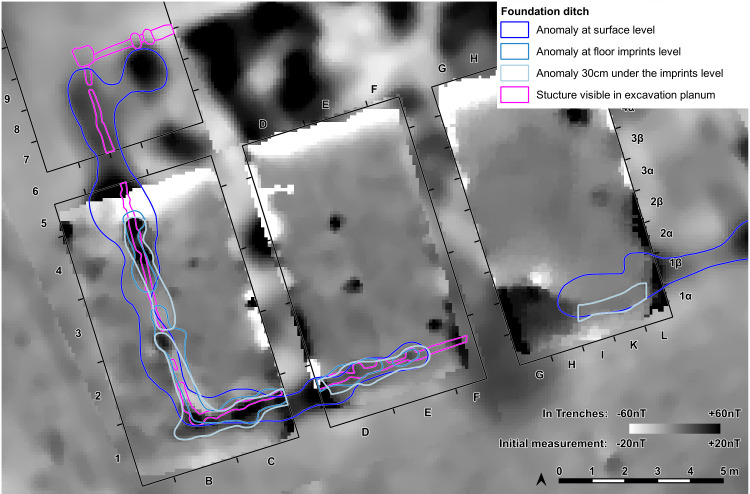
Stăuceni-‘Holm’. **Mega-structure house 5/6. The foundation ditch in the trenches St. 4-5 and St. 43-44, derived from geomagnetic anomalies in the different levels’ measurements and from the actual visible feature.** Made with QGIS. All used pictures made by the author (C. Mischka) and from data acquired/collected by the author (C. Mischka). – Magnetogram. - Layer with excavation grid. - Layer with excavation features.

At the eastern end of trench 44 some clay-imprints showed roundish imprints of smaller twigs. Also, clay with flat rectangular imprints of wooden slabs of maximum 3 cm of width were registered (Fig 14). These could indicate a near internal wall or some internal furniture. No other internal features like walls, ovens or hearths were visible, neither on top of the floor, nor below. This was contradicting the expectations derived from the magnetogram, which suggests a complex internal division with several rooms. Therefore, further geomagnetic surveys were conducted within the three bigger excavation trenches on every new planum, to ensure that no structures were overseen (Figs 15, 16). This technique paid off nicely, as it delivered a clear view of the features under the building’s floor level.

**Fig 17:**
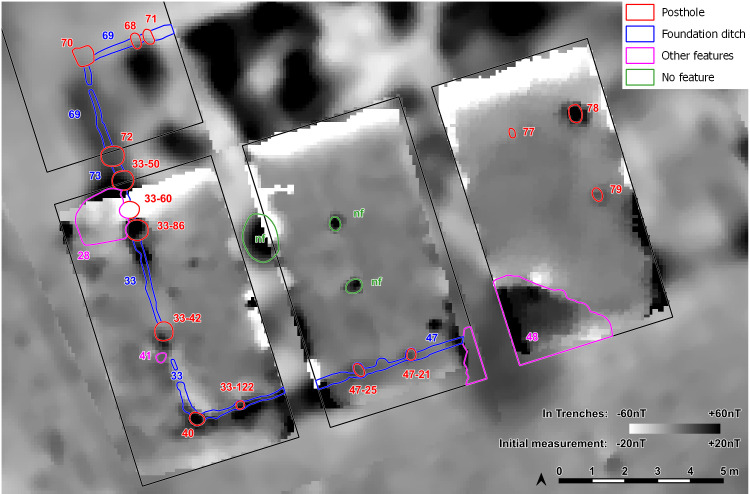
Stăuceni-‘Holm’. **Mega-structure 5/6. Plan with labelled features.** Made with QGIS. All used pictures made by the author (C. Mischka) and from data acquired/collected by the author (C. Mischka). – Magnetogram. - Layer with excavation grid. - Layer with excavation features.

**Fig 18:**
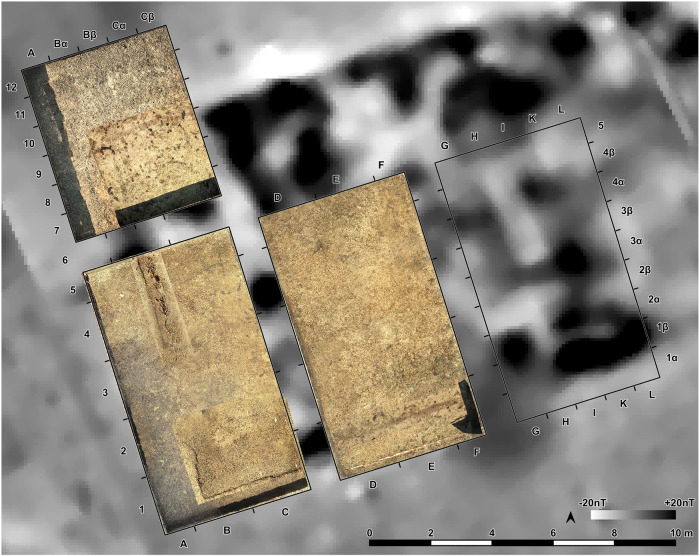
Stăuceni-‘Holm’. **Mega-structure 5/6. Orthomosaic of the foundation ditch, well visible in the C-horizon (planum 6).** The widenings mark the postholes (compare Fig 19). Made with QGIS. All used pictures made by the author (C. Mischka) and from data acquired/collected by the author (C. Mischka). – Magnetogram. - Layer with excavation grid. - Orthomosaics of excavation plana.

The so-called foundation trench (features number 33, 47 and 69) and six pit like features – five underneath the floor (pits number 77–79 and three further anomalies (nf – no feature), and one big, possibly newer pit, disturbing the mega-structure in trench 5 and 44 (feature No. 48) were discovered (Fig 17). The foundation ditch became visible best reaching the C-Horizon as still brownish band in the yellowish soil (Fig 18). In irregular distances, the parallel limits of the ditch were widening, indicating probable posts. Mostly, these widenings had a round shape, but one in the north-west-corner (No. 70) of the building revealed as rectangular.

**Fig 19:**
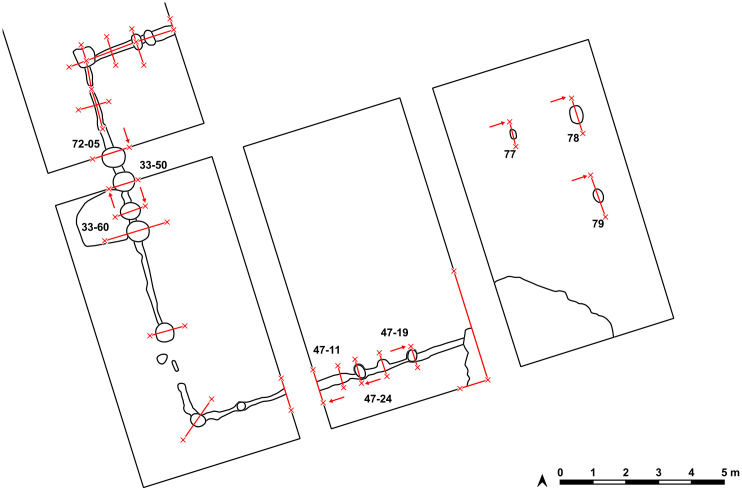
Stăuceni-‘Holm’. **Mega-structure house 5/6. Sections through the foundation ditch and the postholes within the ditch.** Numbered are the sections displayed in Figs 20–22. Made with QGIS. All used pictures made by the author (C. Mischka) and from data acquired/collected by the author (C. Mischka). - Layer with excavation features.

**Fig 20:**
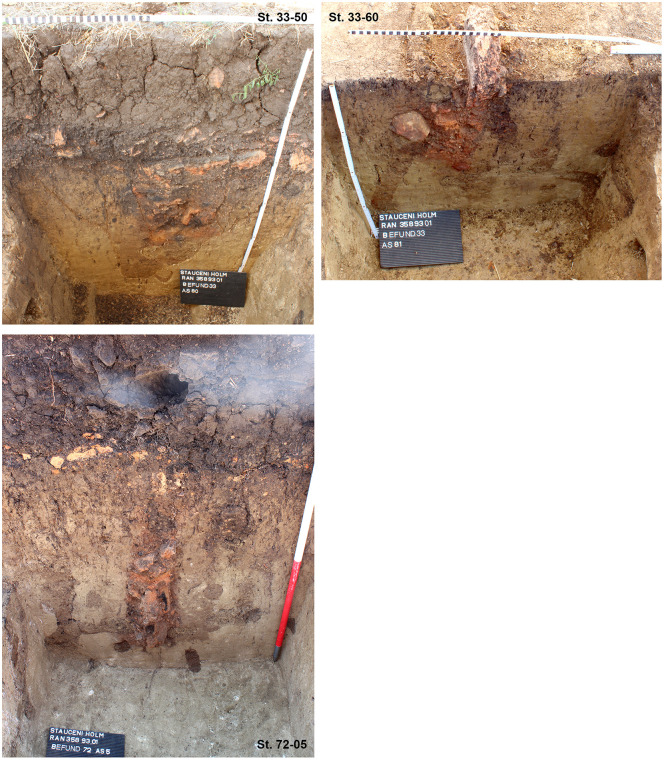
Stăuceni-‘Holm’. **Mega-structure house 5/6. Sections through the foundation ditch and the postholes No. 33−50, 33−60 and 72−05 within the ditch.** For the location of the sections see Fig 19. All used pictures made by the author (C. Mischka): – three photographs of excavation profiles.

**Fig 21:**
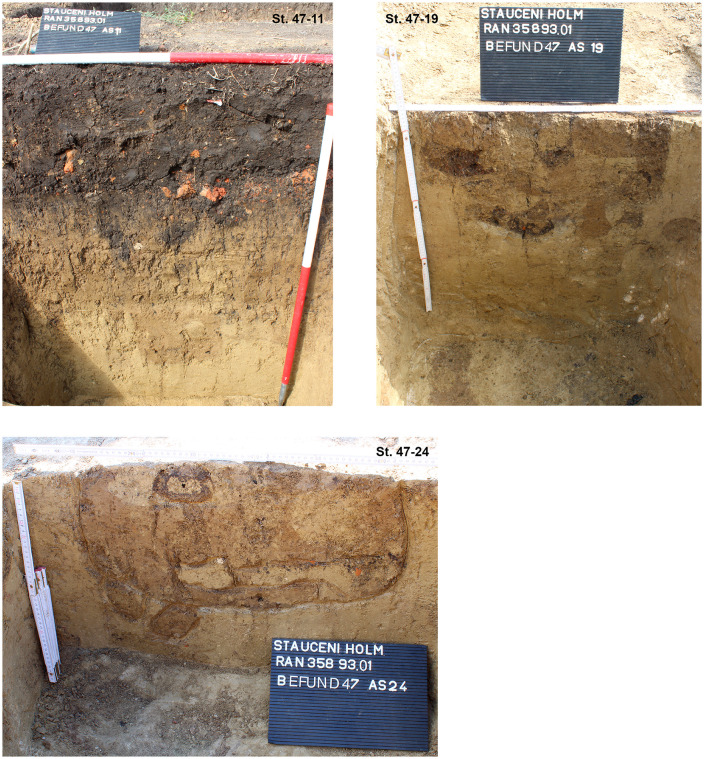
Stăuceni-‘Holm’. **Mega-structure house 5/6. Sections through the foundation ditch and the postholes No. 47−11, 47−19 and 47−24 within the ditch.** For the location of the sections see Fig 19. All used pictures made by the author (C. Mischka): – three photographs of excavation profiles.

**Fig 22:**
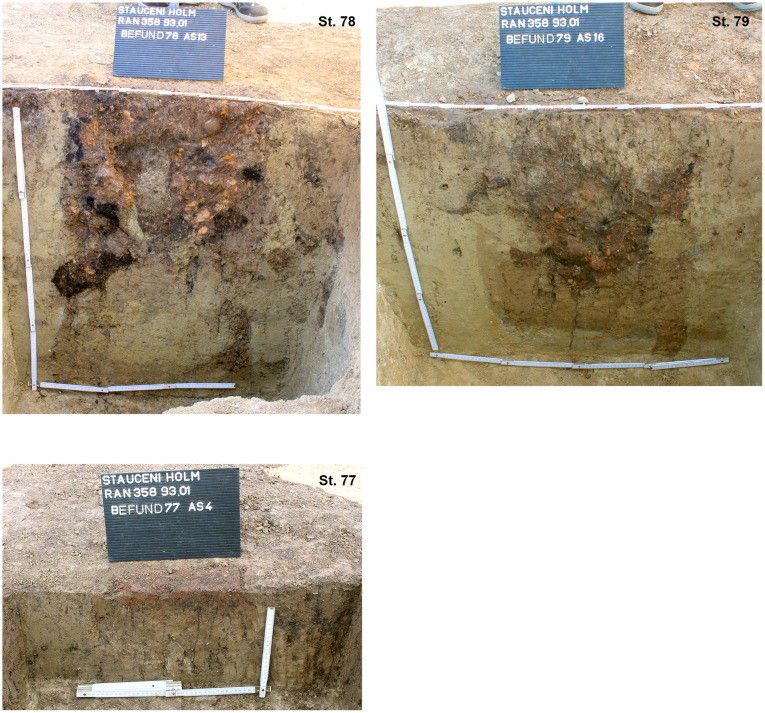
Stăuceni-‘Holm’. **Mega-structure house 5/6. Sections through the pits No. 77-79 in the centre of the mega-structure.** For the location of the sections see Fig 19. All used pictures made by the author (C. Mischka): – three photographs of excavation profiles.

The section profiles were kept for recording of the archaeological layers and the soil horizons. The mainly U-shaped foundation ditch (features number 33, 47 and 69) was partly excavated in negative, partly cut; as well as some of the posts (features number 33–42; 33–50; 33–60; 33–86; 40; 47–13; 47–21; 47–25; 72 and 70) found within the ditch (Figs 17 and 19–21) or underneath or close by the mega-structure (features number 48 (not shown) and features number 77–79) (Figs 17 and 19 and 22). The postholes, features number 78 and 79 in particular, reach a depth of more than 80 cm below the floor of the mega-structure. The section shows perfectly the straight walls and flat bottom of these structures, as well as the filling of the former post with debris of burnt daub and big fragments of charcoals. In feature number 78, a bigger burnt part of the post even remained in carbonized condition.

Near the northwestern corner of the mega-structure, a connected feature was observed, which was labelled “annex” (feature No. 28). It consisted of a concentration of small daub fragments with imprints of small branches. So far, nothing more can be said about its original layout or function.

#### 4.5.3 Samples and finds.

**Soil samples** for extracting **archaeobotanical remains** were taken from the spaces between the imprints of the stems within the floor of the construction as the place with the lowest risk of recent disturbances. The botanical remains (plant macrofossils) deposited in these contexts should also deliver information on the site’s plant economy and on the question, if plant storage, processing or consuming took place within the building. The samples (n = 11; squares B1-B5, C1-C5 and feature 28, square B5 – overall weight 25.30 kg), with an average volume of 2.3 kg from campaign 2023 were wet sieved with water and Soda using sieves with openings of 2 mm and 0,315 mm. Preliminary evaluation and analysis of the carbonized plant remains were conducted in the framework of internship held at the Laboratory for Archaeobotany, Baden-Württemberg State Office for Cultural Heritage. The detailed interpretation of botanical samples of the 2023 campaign will be part of a designated thesis. Within the wet sieved samples, also other small finds like tiny flint debris and small particles from pottery were found but not yet analysed.

The majority of the extracted macrofossils are preserved in carbonized state and only a few mineralized remains were found. The majority of the identified plant macrofossils correspond to remains of crops (badly preserved cereal grains – not further identifiable than as Cerealia) and their weeds (*Polygonum convolvulus*, *Galium* cf. *aparine*, *Chenopodium album, Echinochloa crus-galli*), as well as remains of gathered fruits like cornelian cherry (*Cornus mas*), plums (*Prunus* sp.), elder (*Sambucus* sp.) and hawthorn (*Crataegus* sp.). Carbonized fruit stones can usually be associated with remains of food consumption [48]. In one of the samples, mineralized remains of the ruderal, but also psychotropic/medicinal plant, henbane (*Hyoscyamus niger*), were found. The numerous awn fragments found in several samples most probably belong to feather grass (*Stipa* sp.). This plant is representative of steppe vegetation and has been part of human subsistence in the region since the early Neolithic period [49]. It was probably also gathered and used [50].

**Fig 23:**
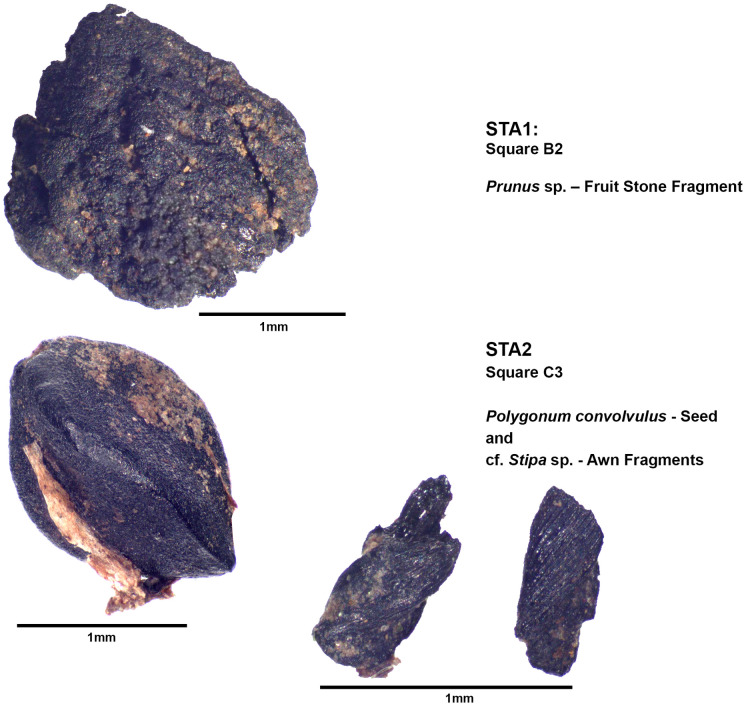
Stăuceni-‘Holm’. ***Mega-structure* 5/6. Samples for** ^**14**^**C-dates.** STA1: square metre B2, *Prunus* sp. fragment of fruit stone; STA2: square metre C3, **A.** *Polygonum convolvulus* seed and **B.** *Stipa* sp. awn fragments. For the location of the finds see Fig 25.

**Fig 24:**
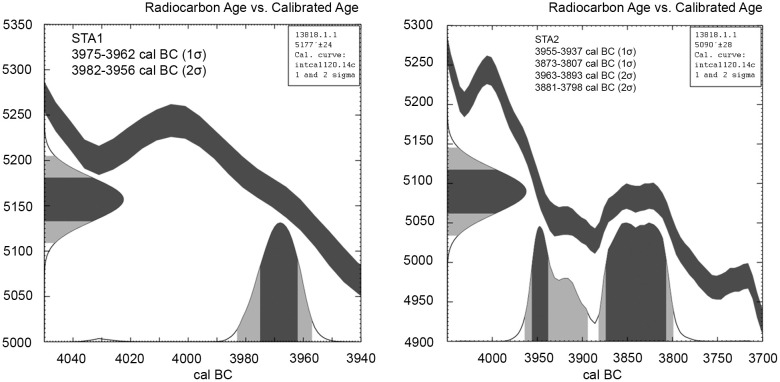
Stăuceni-‘Holm’. ***Mega-structure* 5/6. Curve plot of the calibrated** ^**14**^**C-dates.** Made with Oxcal, by the author (D. Mischka). Oxcal V.4.4, IntCal20 [51].

For ^14^C-dating, two samples were chosen (Fig 23). STA1 from square B2, containing a fruit stone fragment of *Prunus* sp. STA2 from square C3 of a seed of *Polygonum convolvulus,* awn fragments of feather grass and twig fragments. STA1 dates to 3975−3962 cal. BC (1s), while the mixed sample STA2 shows a slightly wider range from 3955−3807 cal. BC (1s) and therewith getting into the next wiggle of the calibration curve, prolongating the age span towards the entire 40^th^ and 39^th^ centuries BC (Table 1; Fig 24).

**Table 1:** Stăuceni-‘Holm’. *Mega-structure* 5/6. ^14^C-dates of the two archaeobotanical samples. For the location of the samples see Fig 25.

AWI nr.	sample label	age (y)	+-(y)	δ^13^C (‰)	BC (1s)	BC (2s)
13810.1.1	STA1	5,157	24	−25.1	3975−3962	3982−3956
13811.1.1	STA2	5,090	28	−26.6	3955−3807	3963−3798

**Fig 25:**
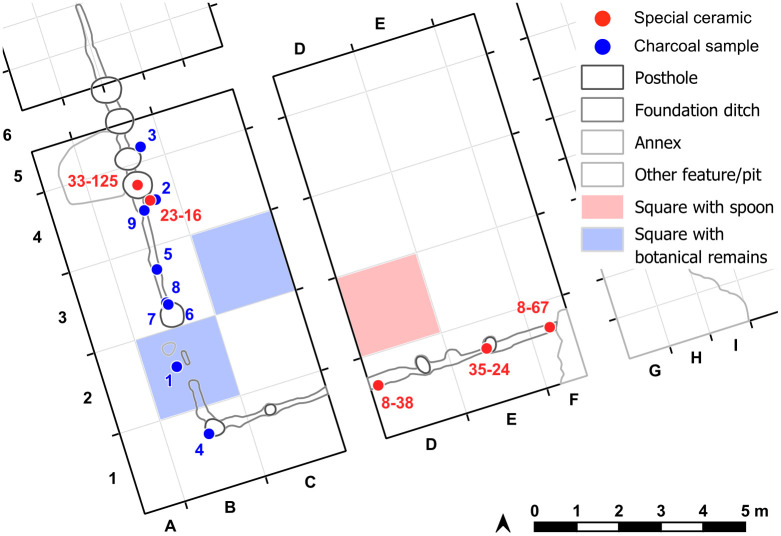
Stăuceni-‘Holm’. ***Mega-structure* 5/6. Plan with the location of the charcoal finds (see** Table 2**) and the dated archaeobotanical samples STA1 from B2 and STA2 from C3 (see** Table 1**).** Made with QGIS. All used pictures made by the author (C. Mischka) and from data acquired/collected by the author (C. Mischka). - Layer with excavation grid. - Layer with excavation features.

Altogether nine samples of **charcoal** from the 2023 campaign were registered and analysed by Sebastian Million from the small foundation ditch and the pit fillings within, as well as from different plana (Fig 25 and Table 2).

**Table 2:** Stăuceni-‘Holm’. *Mega-structure* 5/6. Determination of the charcoal samples from the excavation campaign 2023 by Sebastian Million. For the location of the samples see Fig 25. Anatomical determination of the wood according to https://www.wsl.ch/land/products/dendro/welcome.html; [52].

Number Fig 25	Find number	Determination	Diameter (cm)	Sample condition
1	12-12 (29-53)	no charcoal		
2	23−13	*Quercus*	> 10	1 charcoal, 11 year rings, no rest sample
3	28−22	*Quercus*	2-5	heavily fragmented
4	29-57	indet. LH	2-5	low amount of charcoal
5	29-58	*Quercus*	> 10	low amount of charcoal
6	29-59	*Quercus*	> 10	heavily fragmented
7	29-60	*Quercus*	> 10	heavily fragmented
8^a^	29-62	*Quercus*	> 10	heavily fragmented
9	29-63	no charcoal		
10^a^	29-64	*Quercus*	> 10	heavily fragmented
11^a^	29-65	*Quercus*	> 10	heavily fragmented

^a^Numbers 8, 10 and 11 deriving from the same specimen.

The majority of the charcoal fragments originate from wood with diameter estimated as bigger than 10 cm and belong to oak (*Quercus*). From the two fragments with estimated diameters between 2–5 cm, one belongs to oak and the other specimen is not determinable. These results show good availability of woodland resources at Stăuceni and that oak forests were accessible. In Maidanetske most of the burnt wood was ash (*Fraxinus*, n = 44) but oak was also found (*Quercus*, n = 11) [19], suggesting certain reduced availability of oak and by-passing it with use of elements of the alluvial forests like ash.

As the old wood effect cannot be excluded, AMS dating of the charcoal was not performed for Stăuceni. In a few positions, charcoal was measured and taken as a sample.

**Fig 26:**
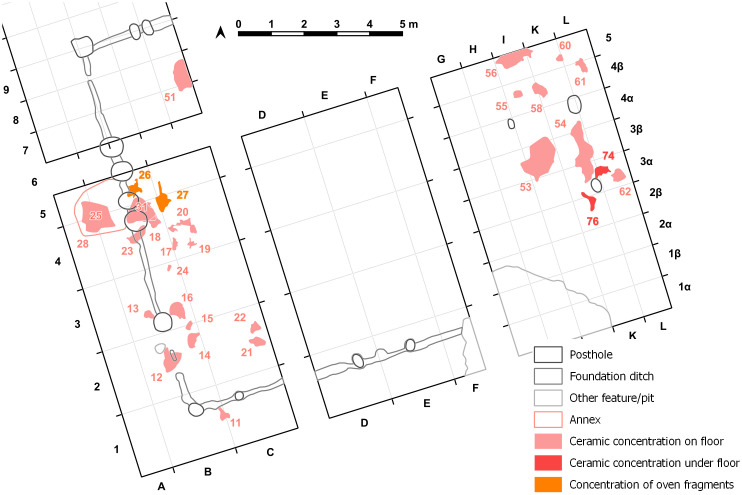
Stăuceni-‘Holm’. **Mega-structure house 5/6. “Annex”, pottery concentrations and oven remains.** Made with QGIS. All used pictures made by the author (C. Mischka) and from data acquired/collected by the author (C. Mischka). - Layer with excavation grid. - Layer with excavation features.

**Fig 27:**
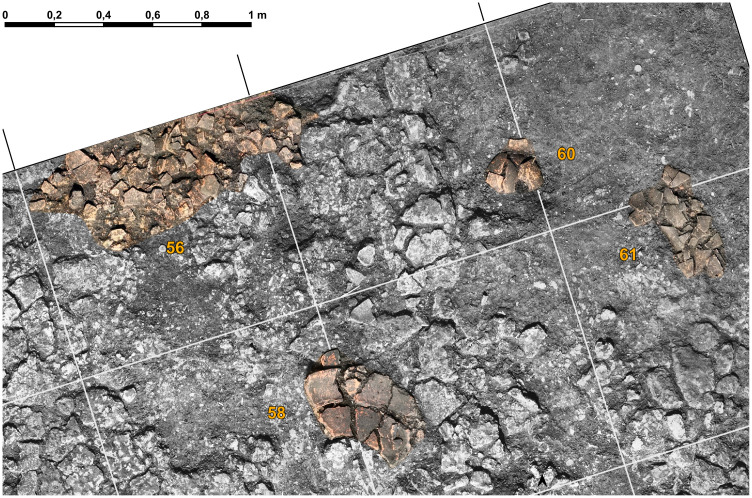
Stăuceni-‘Holm’. **Mega-structure house 5/6. Detail of ceramic concentrations visible in the orthomosaic of trench St. 43, NE-corner.** For the location see Fig 26. Made with QGIS. All used pictures made by the author (C. Mischka) and from data acquired/collected by the author (C. Mischka). - Layer with excavation grid. - Orthomosaic of excavation plana in grayscale. - Layer with excavation features.

**Fig 28:**
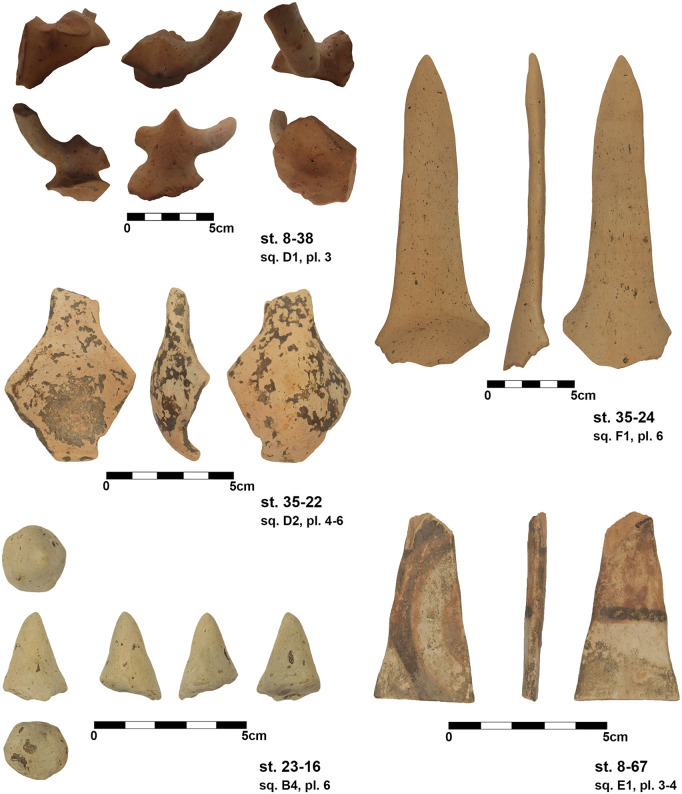
Stăuceni-‘Holm’. **Mega-structure house 5/6. Pottery finds from the internal part of the mega-structure. 2.** For the location see Fig 25. All used pictures made by the author (A. Kovács): – Photographs of 5 different find objects from the excavation.

On the floor of the mega-structure, here and there more or less dense concentrations of **pottery** sherds were registered and recovered (Figs 26–28).

Most of the pottery finds are not yet analysed and will be part of a separate article. Some of the most interesting objects discovered during excavations are zoomorphic protomes. One is quite remarkable, representing a bull’s head attached on a bowl. The horns of the object were massive, but nowadays are broken (Fig 28 st. 8−38). It was found within the daub-layer of the mega-structure. A consistent number of horns was collected from the surface of the settlement site and these are coming from such zoomorphic protomes. A single clay cone was discovered in the excavation, an object common for several Cucuteni A sites, sometimes published under the name of „conical idols”. In reality it is very difficult to assess their purpose (Fig 28 st. 23−16). It was found within a small pottery concentration on the daub*-*layer. Three remarkable ladles were found. One of them still has traces of paint, with different patterns on the two sides (Fig 28 st. 8−67). Only one of the ladles has the cup preserved and this one is oval shaped (Fig 28 st. 35−22). It was found immediately underneath the daub-layer. It is possible that it belongs to an older phase, if it was not moved to this position by modern disturbances. The third object, also found just underneath the daub-layer is a very long handle, more than 15 cm in length (Fig 28 st. 35−24). So far, no anthropomorphic figurine was found within the area of the mega-structure.

**Fig 29:**
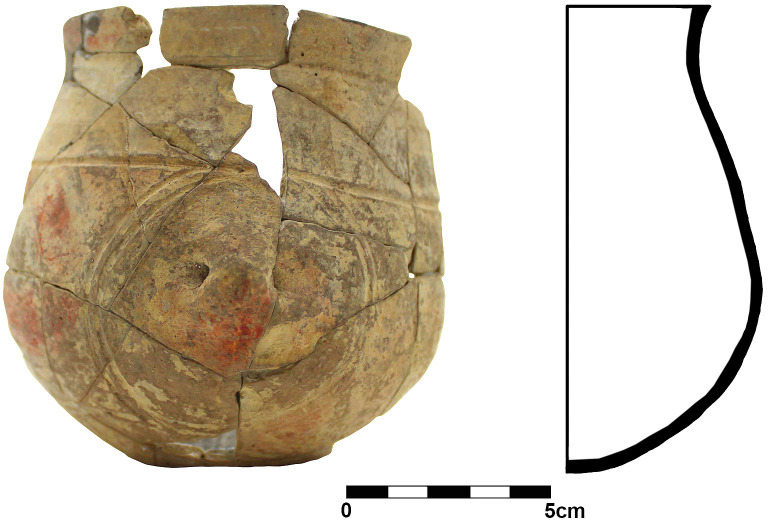
Stăuceni-‘Holm’. ***Mega-structure* house 5/6. Pot No. 33−125 from the bottom of posthole No. 33−86 found 1.10-1.20 m below ground level in the foundation trench.** For the location see Fig 26. All used pictures made by the author (C. Aparaschivei and A. Kovács): – Photography and profile sketch of a refitted ceramic vessel.

**Fig 30:**
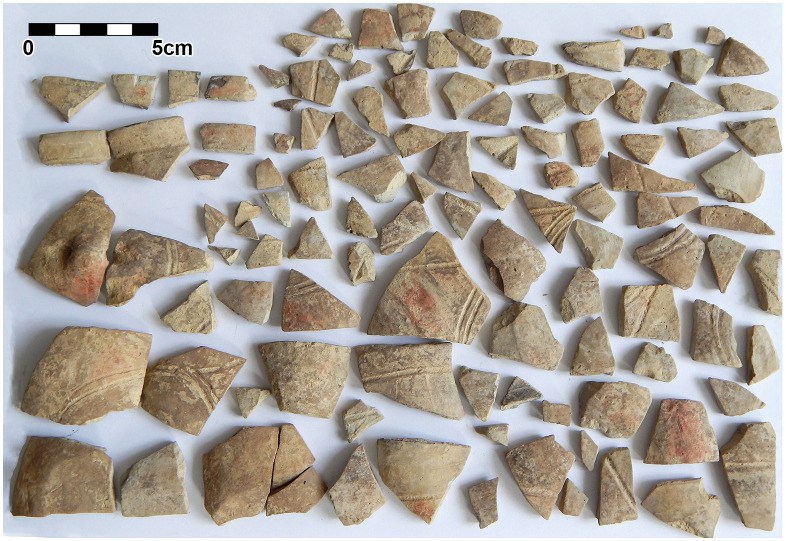
Stăuceni-‘Holm’. ***Mega-structure* house 5/6. Sherds of pot No. 33-125 from posthole No. 33-86 in the foundation trench after cleaning.** All used pictures made by the author (C. Aparaschivei):.

**Fig 31:**
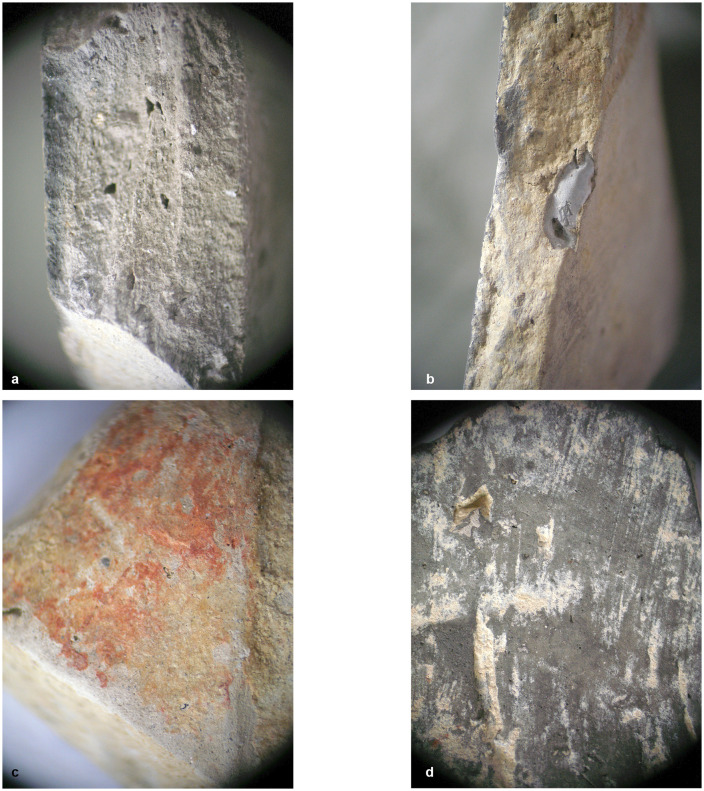
Stăuceni-‘Holm’. ***Mega-structure* house 5/6. Details of the fabrication of pot No. 33-125 from posthole No. 33-86 in the foundation trench.** A – cross section, b – cross section with bone fragment within the paste of the vessel, c – exterior with a layer of red pigments, d – details of the inside of the vessel. All used pictures made by the author (C. Aparaschivei): – Photography of sherd profiles.

Within the foundation ditch and its postholes, almost no finds occurred, except of the features No. 33–60 at the western end of the mega-structure (Figs 17 and 19). Here, burnt daub in a secondary position, as well as reddish, burnt, soft sandstone was used to keep the post in upright position. In the posthole No. 33–86 the highly fragmented sherds of a pot (Find No. 33–125) were discovered (Fig 24). The vessel was found at the bottom of this pit at approximately 1.15 m beneath the modern surface. It is of small size and in an accentuated state of fragmentation (approximately 140 ceramic fragments) (Fig 30). After the restoration of the ceramic materials, it turned out to be a cup of globular shape, with a flared rim, a horizontally perforated protrusion, located on the maximum diameter and a slightly convex bottom (Fig 29). The dimensions are as follows: opening Ø: 9 cm; maximum Ø: 11.8 cm; Ø of the base: 4 cm; H: 10.3 cm; Wall thickness: 0.3–0.5 cm. From the technical point of view, the cup is framed into the category of fine ceramics, being shaped by hand from a clay with very good plastic qualities, mixed with fine sand, few organic materials (chaff and a bone fragment) (Fig 31). The surface was well smoothed, engobed and polished on the outside, the inside being only smoothed. The primary burning occurred in a good reducing atmosphere, resulting in shades of grey on the outside and black on the inside of the vessel (Fig 31). The object was decorated using the following techniques: application, perforation, grooving, incising and painting with red ochre, the pigment being applied only in some areas, after firing the vessel (Fig 31c). Although the shape of the vessel is typical for the ceramic inventory belonging to the Cucuteni A3 culture, the vessel was decorated and fired imitating the usual techniques of the Precucuteni culture, therefore it belongs to the category of Cucuteni ceramics of Precucuteni tradition, with good analogies in numerous settlements researched until today. A nearly identical pot was found in Truşeşti, also dated to Cucuteni A3 [53]. Also, from Truşeşti, another small beaker of highly comparable shape and decoration is known [53].

## 5 Results: the mega-structure at Stăuceni-‘Holm’

### 5.1 The architecture of the mega-structure

As preliminary reconstruction, the construction of the mega-structure can be summarized as follows:

First, a rectangular **ditch** with a minimum width of 15 cm was built to limit the structure. Within the ditch, every 70–90 cm pits were dug to hold a post. The posts were wedged with debris from older buildings, including big fragments of burnt clay from the inside of the mega-structure towards the outside. It is unclear yet, if there was a kind of wall between the posts.

Two **big postholes** (feature No. 78 and No. 79 Figs 17 and 19 and 22 St. 78–79) where found within the mega-structure, probably belonging to the construction as they correspond to “daub-free” round spots within the floor. They are located in the longitudinal centreline of the building. If one more will be found in the northern – not yet excavated – part, a line of three posts would mark the separation into a western and eastern half of the mega-structure. One smaller pit (feature No.77 Figs 17 and 19 and 22 St. 77) for a post was also found, with no correspondence within the excavated trenches, and therefore with a yet unclear function. The other two anomalies (“no feature/nf” in Fig 17) remaining visible in the geomagnetic plan were controlled by excavation, but could not be verified as archaeological features. Instead, they result from an attempt to take out a part of the floor as a block for museal purpose.

Between a line drawn along the big posts (features No. 78 and No. 79) and the eastern section of trench 43, the first fragments of burnt clay appeared, that indicate a **wall** or another kind of installation, for which small branches (wood imprints with round diameters) or slabs (flat rectangular wood imprints) were needed. In this area also much more concentrations of pottery sherds were found, obviously belonging to pots broken *in-situ*, originally placed there on or next to the floor of the mega-structure.

Only in the northwestern corner of the western trench 4 a certain amount of clay with imprints of twigs was found (feature No. 28), but this was located outside the mega-structure and so far interpreted as an **annex**, probably near or connected to an **entrance**. Another feature suggesting a possible access to the mega-structure at this place could be the deep and narrow posts (features No. 33–50, No. 33–60 and feature No. 72) within the foundation ditch and the little pot (No. 33–125 Fig 29) smashed there in the pit. Further, if the hypothesis of a line of three big posts in the centre of the house can be proven, the possible entrance would give access into its northern side.

Within the delimited area, stems from small trees, cut in half were placed on the flat side on the ground, building a **floor** of the mega-structure, keeping free the positions of the posts (feature No. 77, 78 and 79). On top, a layer of clay was placed to smooth the surface. It cannot be decided, if this plaster was originally burned or if it burned later, when the entire construction fell victim to the flames.

Further, the burnt clay is of a small amount, only one layer belonging mostly to the floor plaster. This is different to the typical “normal” houses of Cucuteni sites, where much more daub is found in general. The question remains, if the probable **external walls** were mainly built from wood which burned without a trace, as well as a possible **roof** which at least seems to have existed, supported by posts in the foundation ditch and the posts in the centre of the mega-structure.

Yet, **no internal installations** like ovens or hearths were found, only concentrations of mosaic-like cracked, burned clay registered as a find concentration (features 26–27) in squares B5 and C4-C5 (Fig 26) which is known from substructions of hearths. According to the magnetogram, a big anomaly under the leftover profile between trench St. 4–5 could be a candidate for a hearth or a big pit, but as some comparable anomalies within the house plot disappeared without a trace of archaeological structure in the excavated areas, one should not try to overstress the result of a geomagnetic survey. At least in trench St. 43, remains of a small oven were discovered between the burned daub fragments of the floor, but not recognized *in situ*. But as so far only about 25% of the mega-structure has been excavated, new discoveries in the next campaigns could augment this picture.

### 5.2 About the dating of the mega-structure (Stratigraphy, finds, absolute dating)

For the relative dating of the settlement, the geomagnetic plan shows two house orientations in the southern part of the settlement which *could (but don’t necessarily have to)* belong to at least two different phases. It seems, that the inner ditches/palisades (D4, D6) are overbuilt by the roughly east-west-oriented houses, meaning, the promontory was settled at a certain time, delimited by these ditches and then enlarged towards south. If this hypothesis is correct, the outer ditches D1-D3, together with the palisades D5 could be built to enclose this bigger settlement.

The ceramic fragments of pots and figurines and other clay objects from the entire site and the objects excavated from the mega-structure so far indicate only a single occupational phase, namely Cucuteni A3. But in general, Cucuteni A3 is a quite long phase of 120–300 years, between 4350–4100/4050 cal. BC [42,43], 4350−4200 cal. BC [54]; even 4400−4100 cal. BC [54] or about 4250−4150 cal. BC according to Diachenko et al. [55] for the Upper Siret-Prut region. This time span is long enough, for several construction phases of new buildings within the Cucuteni A3 settlement. All of the clay objects have good correspondence with plastic representations from Scânteia-Dealul Bodeşti, Iaşi county [56], Costeşti, Iaşi county [57], Fulgeriş, Bacău county [58], Drăguşeni-Ostrov, Botoşani county [46,59] or Truşeşti, Botoşani county [60], to mention only a few contemporaneous sites.

Among the material found within the foundation ditch of the mega-structure excavated in the summer of 2023, several daub fragments have been recorded, coming from floors or walls, which were used as wedge-“stones” to keep the posts in the right position. These finds are interpreted as debris from older buildings which were re-used here. They could belong to burned buildings from the inner part of the settlement. If this is the case, the question is to what extent was the inner part of the settlement still in use when the mega-structure was built.

Two samples of archaeobotanical remains, found between the tree-stems of the floor construction are send to the laboratory for radiocarbon dating. Both could be measured and the result dates the building of the mega-structure in the 40^th^/39^th^ century BC.

### 5.3 The function of the mega-structure

Yet, there are no clear suggestions for a reliable hypothesis about the dwelling’s function. So far, relatively few pots were found; only one with a protome of a bull’s head and three fragments of spoons. Only one “conical idol” was found as a special object on the floor of the mega-structure. Among the archaeobotanical finds, few cereals and their weeds, as well as remains of gathered fruits were found. Thus, some crop and fruit consumption could be traced within the building. The presence of the psychotropic plant, henbane, could eventually be related with medicinal or ritual uses, while the feather grass awns could suggest insulation, making mattresses or a range of similar purposes [50]. Only the lithic finds bring some new information, as within the wet-sieved soil samples a steady discovery of tiny flint debris turned to light which could indicate re-sharpening of flint tools or even manufacturing of flint material within the mega-structure. The pottery concentrations at the eastern border of the excavation will perhaps give some more information on the activities within the mega-structure.

### 5.4 Methodology

Regarding the excavation technique, the geomagnetic measurements of every excavated planum revealed that the anomalies, which made us expect internal installations like hearths, platforms or more pits and posts in the construction, were in no way connected to real archaeological structures (at least in the part excavated so far). Only fragments of a probable oven were identified among the burned daub. It became very clear, that most or nearly all of the anomalies resulted from the daub debris from the walls and from the floor plaster in different strength, and are not at all related to such installations. To sum up: contrary to other beliefs, **no information on the interior layout can be derived from the magnetogram!** After the burnt daub was taken away, the anomalies disappeared nearly completely, only the foundation ditch remained, while on the other side new structures – postholes – appeared.

Regarding the archaeobotanical analysis, the sample volumes are very small and the concentration of plant remains is very low. But, even if the samples cannot be taken as representative, varied remains from crops, weeds, gathered and other plant remains were found, giving a meaningful insight in the activities within the mega-structure.

## 6 Discussion

Although the excavations in Stăuceni-‘Holm’ have not yet been completed, we can confirm several conclusions and previous observations [4,16,19,26,29] and add some of our own.

### 6.1 Location and age of the mega-structure

The mega-structure at Stăuceni-‘Holm’ is located at the western edge of the distribution area and, according to the pottery typo-chronology, dated to the Cucuteni A3 phase. This means that, according to the typology of the pottery, it is younger than the closest Pre-Cucuteni site Baia from the Suceava district, but older than the other sites excavated in Ukraine and on the Romanian-Moldavian border.

Nevertheless, the absolute dates of the 40^th^/39^th^ century BC contradict the actual chronological position of Cucuteni A3, which is said to be clearly older than 4000 cal. BC with a beginning at 4350 and an end at 4230 or 4050 cal. BC the latest [42,43,53–55]. For the 40^th^/39^th^ century BC, Cucuteni AB-material should be expected.

It is questionable whether the dates are reliable. But the samples were taken from very well protected locations between the floor boards and, therefore, as safe as possible from disturbances caused by modern ploughing or by bioturbation, from a clear stratigraphical position, which we consider to be connected to the period of construction of the mega-structure. Furthermore, the samples belong to short-living, annually growing plants, so an old wood effect can be excluded. Therefore, at this stage of research, we have to rely on these absolute dates.

The connections of the absolute dates to the Cucuteni A3-pottery, on the one hand, and of these finds to the construction of the mega-structure, on the other hand, is also given, as the prototypical Cucuteni A3-pot (No. 33–125 Fig 29) was found within the foundation ditch of the mega-structure and therefore connected to the construction and to the ^14^C-dates.

Of course, a renewal phase of the floor can be taken into account. Then, also an offering of an “old pot” from the Cucuteni-A3 settlement placed in the ditch can be discussed, as also older daub fragments were used to wedge the posts within the ditch. But if so, the post(s) within the ditch must have been renewed as well. These posts are most probably necessary as carriers of the roof. That is why this entire scenario would mean a complete re-build of the mega-structure, which then was filled up again with Cucuteni-A3 pots found on the floor. Altogether, this idea is refused at this scale.

But, if we can trust the absolute dates and they date the construction of the mega-structure in the 39^th^/40^th^ century and the finds belong to the date, then, the absolute chronology of the Cucuteni A3 phase (and probably the entire chronology) can be doubted. Diachenko et al. [55] (like others) realized that there are some discrepancies within the absolute dating of the Cucuteni and Trypillia phases and they offer some ideas for the solution of the problem, related to different developments in different regions with core, periphery and irregular distribution patterns of the materials, depending of early, late or piecemeal adopters [55]. For Cucuteni A3 for example, the time span differs in length (from about 100 years to more than 300 years) and in the starting and ending time (from shortly after 4400 cal. BC to about 4000 cal. BC) [55]. Nevertheless, all dates they took into account are still older than 4000 cal. BC.

Of course, the actual typochronology is relying often on a very low number of ^14^C-dates within the different regions, contending old dates with long standard deviations and often no clear connection to the finds they were taken from (wood, archaeobotanical remains, bones etc.) or those of unclear connection to typologically significant pottery. For the Upper Siret-Prut region, [55] supplement] only four ^14^C-dates could be taken into account, three derived from charcoal from Drăguşeni-Ostrov with a high standard deviation of 100, and only one precise date from a bone from the site of Preuteşti-Haltă, all measured before 1995. So, without the methodologically problematic charcoal dates, the entire region is dated by only one date from a pit from one site.

At least from the mega-structure at Ripiceni-‘Holm’, another ^14^C-date is published, as already mentioned, which is very interesting here, because according to the pottery typology the site is dated to Cucuteni AB1. The calibrated date of 4041−3975 cal. BC (1 sigma) is fitting to the proposed absolute age of this phase (~4000 - ~ 3800 cal. BC according to Diachenko et al. [55]). These ^14^C-dates would be more or less contemporaneous to the mega-structure of Stăuceni-‘Holm’. For Nebelivka and Dobrovody, the absolute time span for the mega-structures starts also in the 40^th^/39^th^ century. But for both sites, the mega-structure could also belong to the 38^th^ century, the same as Maidanetske mega-structure Number 3.

As an interim conclusion, it can be stated that more absolute dates of good contexts are desperately needed. Only with a good independent absolute chronology it will be possible to understand the typochronological development of pottery styles per region and the role of mega-structures. Furthermore, only on this basis, a serious discussion on mobility, early or late adopters and so on is possible. According to the actual state of research, Cucuteni A3 would have occurred first in the east and distributed later towards the west, an idea which contradicts the common opinion [55].

### 6.2 Foundation ditch

At Baia, a very similar foundation ditch, also of more than 1 m in depth, was discovered by excavation. In the drawing of the sections, the ditch and the post-holes are directly underneath the floor-level of the mega-structure [29]. On the published photos, the post-holes and even the remains of the posts are clearly visible in the profiles, but not visible as features on the planum. Obviously, they have been detected while cutting the mega-structure. Unfortunately, the position of the profiles is not indicated on the plan [29]. Nevertheless, the pits and postholes look more or less identical to Stăuceni-‘Holm’. Baia is dated according to the finds to an early Pre-Cucuteni (Pre-Cucuteni I late or Pre-Cucuteni IB according to Aparaschivei [27]).

No foundation ditches were described in either Nebelivka or Maidanetske. There have been no reports published about the geomagnetic measurements of the different excavation layers or the profiles of the areas where the ditch must have been; therefore, it is not clear to us whether such surrounding ditches could have existed. Chapman et al. would also not rule out the existence of a ditch in Nebelivka [22]. But, according to Gaydarska et al., a construction on granite rockhead is mentioned for Nebelivka, which could mean that a foundation ditch would have been carved in the rock, and therefore it would have been found during excavation, if it had existed [5,61].

In Maidanetske, pits and older layers have been reported underneath the mega-structures, according to the published profile directly underneath the floor and daub layer [19]. However, no profiles crossing the outer limits of the megastructure in search of ditches or other wall-related installations have been published. According to our observations in Stăuceni-‘Holm’, it was necessary to eliminate about 20–30 cm of sterile-looking sediment in order to see the ditches. Without the geomagnetic measurements, there would have been nothing suggesting their presence and no reason to continue the excavation this deep.

### 6.3 Internal anomalies

One important fact is that the anomalies seen in the topsoil on the magnetogram of the mega-structure were “fake”, caused only by the varying strength of the collapsed structure’s daub. This discovery is important in view of the opinion of Hofmann et al., who use the presence or absence of internal anomalies for a classification of the mega-structures [19]. Nevertheless, the magnetic plans indicate the presence of burnt daub to a certain amount in some of the mega-structures. The majority of these anomalies are *not* caused by pits, posts, hearths or clay bins etc. The amount of daub can only indicate the use of daub for the floor, the walls or even the roof. On the other hand, the absence of the internal anomalies in some of the mega-structures could reflect the state of preservation of the original features, independent of former internal divisions, walls or roofs. Perhaps at least some of the mega-structures without visible internal anomalies are just more heavily destroyed by erosion, so that no bigger burnt daub clusters survived and only the deeper ditches are still clearly visible in the geomagnetic survey. The discoveries from Stăuceni-‘Holm’ should be a warning for everybody, to be cautious when interpreting the geomagnetic results without excavation.

### 6.4 The mega-structure – one feature or a composition?

In Stăuceni-‘Holm’ it will be possible to check through the ongoing excavation if the floor is found within the entire anomaly of the geomagnetic plan of the mega-structure. So far, this is the only mega-structure where such a floor has been preserved. This will yield further information about whether the mega-structure is one feature or perhaps composed of several parts with different constructions – like roofed parts and courtyards. Further, it can show whether the floor was built all at once. This could be a prove against the hypothesis that the big rectangular geomagnetic anomaly is caused by two or more buildings, closely related, and not one big construction [28].

### 6.5 Finds

Apart from the pot No. 33–125 (Fig 29) found in the post hole (No. 33–86), the finds from the internal part of the mega-structure are in congruence with the sparse finds found in the other excavated mega-structures – except Baia, even if not yet researched in detail. No grinding stones have been found, only a few carbonized remains of plants, including cereals, their weeds, as well as fruits. Anthropomorphic or zoomorphic statuettes are missing; only a horn-protome attached to a pot and a so called “conical idol” were found. There have been found 87 flint artefacts, about half of them burned, researched by Aurora Botsch in her MA-thesis. Their number is higher than in Maidanetske (1 flake, 3 artificial debris) or Nebelivka (6 artefacts) [26]. Of course, this is not surprising, as in the Ukrainian sites the nearest sources for lithic raw materials are several hundred kilometres away. In Baia, an exceptional find were the 200 vessels, of which 25 specially decorated. Among the clay finds, several fragments of zoomorphic figurines or vessel protomes came to light. The quantity of flint is not published so far, but, at least, the lithic artefacts are mapped in the plan of the mega-structure [29].

Therefore, at the actual state of the research it seems unrealistic to consider the function of the building as storage building or a communal place for consumption of food. Also, there are no clear indications for cult purposes. Perhaps, the mega-structure was just a bigger house for a bigger family, a communal building for decision making or a meeting place for special high-ranking inhabitants reflecting a change towards a more hierarchized organization of the community (see [62]).

## 7 Significance of mega-structures in Pre-Cucuteni and Cucuteni A contexts?

Hofmann et al. [19] propose to interpret the mega-structures as elements of the political, economic and social organization of mega-sites. They differentiate two kinds of mega-structures. First, there are those of greater dimensions in positions P1 and P2 on the central plaza, in the north-east quarter of the mega-site, perhaps related to a main entrance leading to the centre of the mega-sites (Fig 2). And the other types of mega-structures are most often related to the ring-corridors (P3), within other pathways (P4) from outside towards the inside of the mega-site or in front of the outermost ring.

Of course, Stăuceni-‘Holm’ is not a ring-settlement and not a mega-site, but a site with ditch-systems, as usual in the Cucuteni and Pre-Cucuteni sites. The oldest mega-structure so far is known from a Pre-Cucuteni I late/IB context at Baia. But then, depending on the reliability of the absolute dates, Stăuceni-‘Holm’ is, together with Ripiceni-‘Holm’, the second oldest mega-structure; or perhaps contemporaneous to Nebelivka and Dobrovody, all dated to the 40^th^ and 39^th^ century BC. But Nebelivka, Dobrovody and Maidanetske No. 3 could also have been built in the 38^th^ century BC. However, according to the finds, the Cucuteni-A3 style from Stăuceni-‘Holm’ is older than the Cucuteni AB1 of Ripiceni-‘Holm’.

In Baia, the mega-structure is situated more or less in the centre of the settlement, which can be compared to the special position inside the plazas (P1). In Stăuceni-‘Holm’, the mega-structure is situated between the ditches, as in Ripiceni-‘Holm’, but parallel to them, not perpendicular, like in Ripiceni-‘Holm’. However, it is yet unclear, if the ditches are contemporaneous at both sites. The mega-structure would have been clearly visible in Stăuceni-‘Holm’ only if the smaller, neighbouring houses were not contemporaneous. In Ripiceni-‘Holm’, the mega-structure is in a more isolated position, with unbuilt space around it, according to the magnetogram. As Stăuceni-‘Holm’ is located on a promontory, the mega-structure is at least in the vicinity to the entrance, which, because of the deep slopes to the west, north, and east, must have been on the south side of the site. If the ditches as a feature are comparable or “substitutional” to the outer most row of houses of the mega-sites, the Stăuceni-‘Holm’-mega-structure is positioned like P5 in the mega-sites: outside the main part of the settlement and well visible for people approaching the place, and not like P4, oriented towards the centre along the pathway.

According to the finds of burnt daub, which are typical in normal houses, in a secondary position, wedging the posts in the foundation ditch, the megastructure can be seen as the late (perhaps the latest?) phase of the site. For the organization of a settlement that is quite small in comparison to the mega-sites (approx. 320–350 inhabitants, with 7 persons/household), the mega-structure seems not necessary, but, as mega-structures belong to a regular feature of Cucuteni-Trypillia sites, it is still the best candidate for the government of such settlements. And, they were available as an option since early in the Precucuteni I times, as Baia proves. Ohlrau and Rud stressed already the presence of mega-structures as an integral part of smaller sites too, following a similar planning principle to that of the mega-sites [23]. It has to be discussed further, whether the inhabitants found a long-lasting tool for aggrandizing settlements and to live in huge user groups through a sort of modular organizational structure related to the mega-structures (compare [63]).
